# Transcriptome analysis and metabolic profiling reveal the key role of carotenoids in the petal coloration of *Liriodendron tulipifera*

**DOI:** 10.1038/s41438-020-0287-3

**Published:** 2020-05-01

**Authors:** Zhaodong Hao, Siqin Liu, Lingfeng Hu, Jisen Shi, Jinhui Chen

**Affiliations:** Key Laboratory of Forest Genetics and Biotechnology of Ministry of Education, Co-Innovation Center for Sustainable Forestry in Southern China, Nanjing Forestry University, Nanjing, Jiangsu China

**Keywords:** Gene expression analysis, Metabolomics, Non-model organisms, Plant development

## Abstract

*Liriodendron tulipifera*, also known as tuliptree, is a popular ornamental horticultural plant with extraordinary tulip-shaped flowers characterized by an orange band near their base. The mechanisms underlying petal band-specific pigmentation during *L. tulipifera* flower development are unclear. Here, we combined nontargeted and targeted metabolomics and transcriptomics to identify a pathway cascade leading to carotenoid biosynthesis that is specifically activated in the petal band. The comparative analysis of carotenoid metabolites between *L. tulipifera* and *Liriodendron* hybrids indicates that γ-carotene, a rare carotene in plants, is the most likely orange pigment responsible for the coloration of the petal band. Phenotypic and transcriptomic analyses of developing petals reveal that the band area is first predefined by the loss of green color. Later, the band is maintained by locally activating and repressing carotenoid and chlorophyll biosynthesis genes, respectively. Two rate-limiting genes of carotene biosynthesis, carotenoid isomerase (*CRTISO*) and epsilon lycopene cyclase (*ε-LCY*), encode the core enzymes responsible for petal band-specific orange pigmentation in *L. tulipifera*. In particular, a putative additional *ε-LCY* copy specific to *L. tulipifera* may contribute to the distinct petal coloration pattern, compared with *L. chinense*. Taken together, our work provides a first glimpse of the metabolome and transcriptome dynamics in tuliptree flower coloration and provides a valuable resource for flower breeding or metabolic engineering as well as for understanding flower evolution in an early woody angiosperm.

## Introduction

Petal color is a major floral trait involved in attracting pollinators to ensure reproductive success. Phenotypic changes in petal color can cause shifts in flower–pollinator interactions and thus drive reproductive isolation and even speciation^1,2^. Flower color diversity is mainly defined by three major pigment types: flavonoids, carotenoids and betalains^3^, and the precise spatiotemporal regulation of the expression of these pigment biosynthesis-related genes generates specific coloration patterns^4^. The first two pigments are widespread across the plant kingdom, while betalains are found only in the Caryophyllales^5^. Among the plant pigments, flavonoids are arguably the best-characterized plant secondary metabolites and exhibit the widest color range, conferring orange to blue (anthocyanins) and pale-yellow (chalcones, aurones and anthoxanthins) coloration^6^. In particular, cytosol-synthesized and vacuole-localized water-soluble anthocyanins are the main flavonoid group and contribute to color pigmentation in many flowers^7^. The anthocyanin biosynthesis pathway and its regulation have been well characterized, showing that a highly conserved “MBW” regulatory complex consisting of R2R3-MYB, basic helix–loop–helix (bHLH) and WD40-repeat (WDR) proteins is of central importance^8^. Although the biosynthesis pathway of carotenoids, a class of plastid-synthesized and localized lipid-soluble C40 tetraterpenoids, has been well established, the underlying transcriptional regulatory mechanisms are less well understood, especially in flower pigmentation^9–11^. Carotenoids are not only indispensable to plants, showing functions in pigmentation^10^, photoprotection during photosynthesis^12^, and the biosynthesis of the phytohormones abscisic acids and strigolactones^13,14^, but are also critical to human nutrition and health^15^. The identification of the regulatory mechanisms underlying carotenoid biosynthesis and accumulation is urgently needed, which may aid in breeding ornamental plants with improved color traits and agricultural crops with improved nutritional quality^9,10^.

Carotenoid accumulation and pigmentation are largely determined by the differential expression of carotenoid biosynthesis genes^16^. Recent studies have indicated that various transcription factors (TFs) in diverse species (Supplementary Table 1), including the bHLH TF PHYTOCHROME-INTERACTING FACTOR 1 and the basic leucine zipper TF LONG HYPOCOTYL5 (HY5) in *Arabidopsis* seedlings, the APETALA2/ethylene-responsive element-binding protein TF RAP2.2 in *Arabidopsis* leaves, two R2R3-MYB TFs (CrMYB68 in mandarin flavedos and AdMYB7 in kiwifruit fruits), and the B-box zinc-finger TF SlBBX20 in tomato fruits, can directly regulate one or more components of the carotenoid biosynthesis pathway and thus affect carotenoid accumulation in plants^9–11^. However, several MADS-box ripening regulators that can regulate carotenoid biosynthesis genes do not dramatically affect flower petal color in tomato^11^, suggesting that plants are more likely to have evolved different regulatory mechanisms for carotenoid biosynthesis during flower coloration. Knowledge regarding the transcriptional regulation of carotenoid biosynthesis genes during flower pigmentation is still limited. For example, the F-box protein coronatine insensitive 1 can affect the β-carotene content in the tobacco floral nectary, probably through the R2R3-MYB gene *MYB305*, although there is a lack of direct experimental evidence^17^. In addition, the R2R3-MYB gene *reduced carotenoid pigmentation 1* (*RCP1*) positively regulates carotenoid biosynthesis in a nectar guide pattern during flower development in monkeyflowers^18^. Encouragingly, substantial progress has recently been made towards the regulation of floral carotenoid pigmentation by screening *Medicago truncatula* insertion mutants for the loss of carotenoid-derived pigment accumulation in petals^19^. Specifically, the R2R3-MYB TF WP1, together with the bHLH protein MtTT8 and the WDR family member MtWD40-1, forms an “MBW” complex that promotes floral carotenoid accumulation and pigmentation by directly activating the expression of carotenoid biosynthetic genes, including *lycopene ε-cyclase* and *lycopene β-cyclase*, in *Medicago* petals^19^.

*Liriodendron tulipifera*, belonging to the *Liriodendron* genus of the monogeneric subfamily Liriodendroidae (Magnoliaceae family), is native to eastern North America. There are only two species in this genus, with the other being *Liriodendron chinense*, native to East Asia, in accordance with a classic intercontinental disjunct distribution^20^. These two closely related forest trees are significantly different from other magnolia plants and are morphologically very similar, although they diverged ~10–16 million years ago^21^. There are several distinctive morphological characteristics between these two *Liriodendron* species, one of which is flower color. The petals of *L. chinense* are green throughout with yellow veins, whereas *L. tulipifera* has a bright orange band near the base of the petals^22^. Moreover, interspecific hybridization between these two species is possible^23^ and *Liriodendron* hybrids are characterized by larger flowers in which most of the petal surface is colored by orange pigments and significant interspecific heterosis in biomass^20^. In addition to its high commercial value in timber and honey production^24^, *L. tulipifera* has been cultivated as a popular ornamental horticultural plant for parks and gardens^25^. Although its extraordinary tulip-shaped flowers with a colored band at the base are a defining horticultural trait of *L. tulipifera*, the developmental, regulatory and biochemical basis of petal band coloration is unclear. Here, we combined transcriptomics and metabolomics across *Liriodendron* species to identify the main pigments contributing to orange band formation and define a plausible transcriptional regulatory mechanism.

## Results

### *Liriodendron tulipifera* petal development and coloration

The fully developed flowers of *L. tulipifera* are characterized by a broad orange band on the petals. To characterize the morphological trajectory that generates the characteristic flower shape and color, we dissected flower buds or flowers in six different stages (Supplementary Fig. 1 and Supplementary Table 2). In the flower buds with an indehiscent bract (stage I) or a just dehiscent bract (stage II), a predefined band area of the petal with no obvious coloration is apparent, while the upper side of the petal has started to turn green. When flower buds progress to stage III or IV, the bract becomes fully senescent or abscised. Additionally, the whole flower bud becomes soft and starts to produce nectar. The band of petals shows a similar light yellow coloration in these two stages. In the next stage, the sepals fully expand, the petals remain stuck together in the flower full of nectar, and the band of petals becomes orange. Finally, in stage VI, the sepals bend downward, the petals are fully expanded, and the petal band turns dark orange. At this last stage, fertilization is complete, and the flower senesces. Notably, the width of the petal band seems to remain constant in the developing petals with an increasing size (Supplementary Fig. 2). However, considering the similar coloration between stages I and II, III, and IV (Supplementary Fig. 3), we ultimately sampled petals from four flower stages (II, III, V, and VI), to examine the developmental time course of tuliptree petal coloration for subsequent transcriptomic and metabolomic analyses. In addition, the sepals stayed green and exhibited no noticeable change in color compared to the petals during tuliptree flower development (Supplementary Fig. 1). Orange coloration is nonuniform across the surface of petals with a band-specific pattern near the base of the petals (Supplementary Fig. 1). Therefore, to better explore the potential molecular mechanisms underlying tuliptree petal coloration, we also conducted two sets of comparative studies, examining petals versus sepals and the lower side versus upper side of the petals at the second-to-last stage.

### Global metabolomic changes during *L. tulipifera* flower development

To profile the metabolic changes during *L. tulipifera* flower development, we performed nontargeted metabolome analysis using liquid chromatography-mass spectrometry (LC-MS). We detected and identified 4883 and 6952 metabolites in the positive and negative ionization modes, respectively, across all samples (Supplementary Table 3). Among the metabolites, 4373 and 5752 were annotated with definitive formulas, while the rest received similar annotations (Supplementary Table 3). The quality of the nontargeted metabolome data was good, as evidenced by the high correlation among QC samples (Supplementary Fig. 4) and the PCA results of the time-course and two comparative analyses (Supplementary Fig. 5).

In the time-course dataset, there were a total of 4799 metabolites, consisting of 1825 and 2974 metabolites from the positive and negative ionization modes, respectively, that showed a significant difference (*p* < 0.05) between at least two stages (Supplementary Table 4 and Supplementary Fig. 6). Five clusters were identified based on all significantly differential metabolites during *L. tulipifera* petal development using the *K*-means clustering method (Supplementary Fig. 7). Clusters A and B exhibited a general downward trend, whereas clusters D and E exhibited a general upward trend. More specifically, the metabolite contents changed gradually in the first three stages, but decreased or increased dramatically towards the last stage in clusters B and D, respectively. Conversely, clusters A and E presented a dramatic decrease and increase, respectively, in the first three stages, after which the trend became flat. Kyoto Encyclopedia of Genes and Genomes (KEGG) enrichment analysis showed that three flavonoid-related pathways: “phenylpropanoid biosynthesis,” “flavonoid biosynthesis,” and “flavone and flavonol biosynthesis” were significantly overrepresented in cluster A (Supplementary Fig. 7). Since anthocyanins are a class of flavonoids derived from phenylalanine, this type of major plant pigment may not be the major determinant of *L. tulipifera* petal coloration. Notably, the “glycolysis/gluconeogenesis” pathway was overrepresented in cluster A, whose product d-glyceraldehyde 3-phosphate (glyceraldehyde-3P) is used as the substrate for the “terpenoid backbone biosynthesis” pathway, which was enriched in cluster E. Considering that one of the three major plant pigment types, the carotenoids, is a subclass of terpenoids and that the other type, the betalains, are limited to the order Caryophyllales^6^, we speculate that carotenoids may play a critical role in *L. tulipifera* petal coloration, which is also consistent with the physiological measurement results^26^.

According to the two comparative datasets, there were 695/868 (positive/negative) and 921/1216 (positive/negative) metabolites that were significantly different (*p* < 0.05) between the petals and sepals (Supplementary Table 5) and between the lower side and the upper side of petals (Supplementary Table 6), respectively, in developmental stage III. KEGG enrichment analysis showed that metabolites that differed between petals and sepals were enriched in flavonoid-related pathways (Supplementary Fig. 8a), while those that differed between the lower and upper sides of the petals were enriched in terpenoid-related pathways (Supplementary Fig. 8b).

### Global transcriptomic changes during *L. tulipifera* flower development

**Fig. 1:**
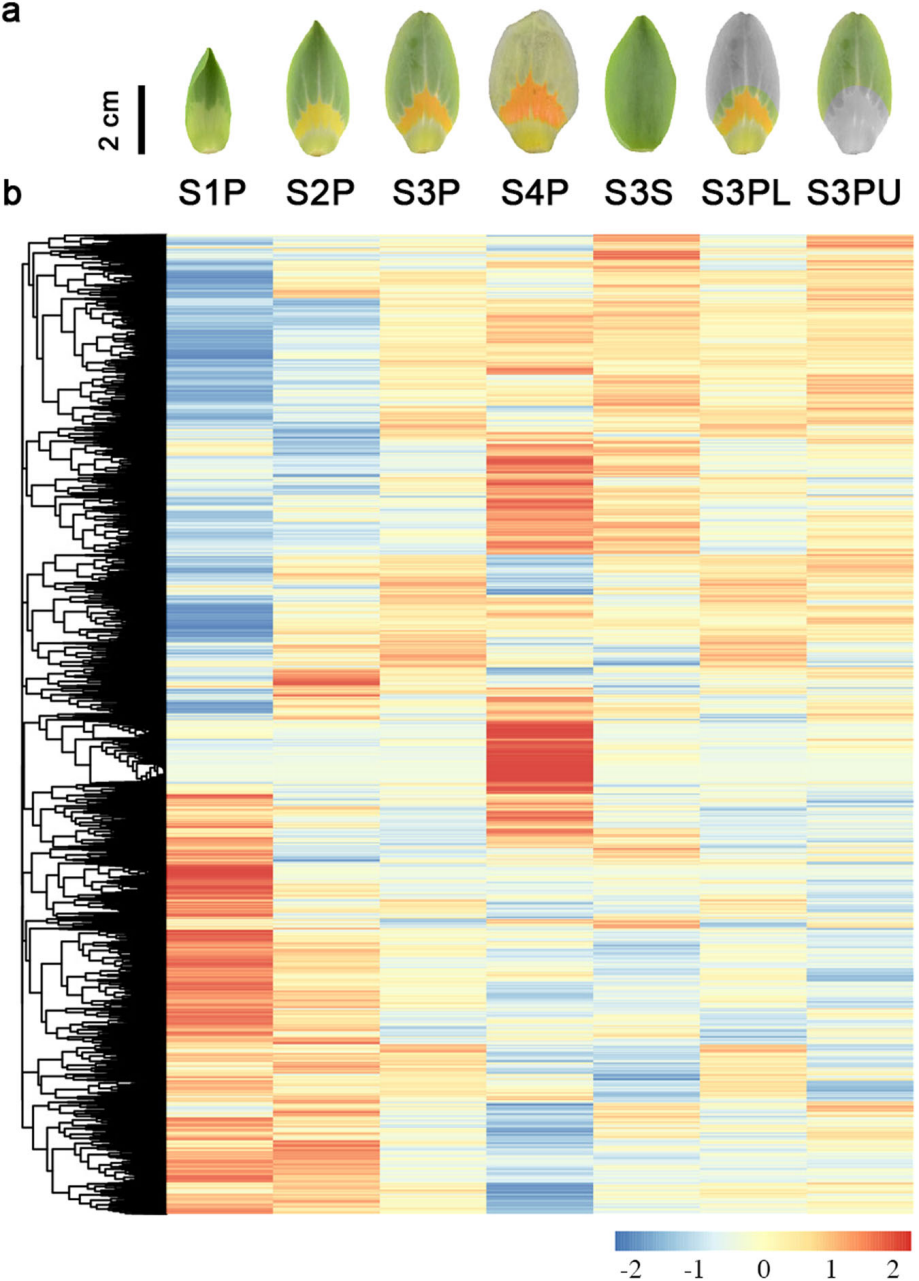
Transcriptome analysis of petal development in *L. tulipifera*. **a** Petal samples at four developmental stages (S1P, S2P, S3P, and S4P), a sepal sample at the third developmental stage (S3S), and the lower side (S3PL) and upper side (S3PU) of a petal sample at the third developmental stage. **b** Hierarchical clustering of unigene expression. The detailed sample and developmental stage selection procedures are included in Supplementary Fig. 1

RNA-sequencing (RNA-Seq) was used to profile genome-wide gene expression and transcriptome changes during *L. tulipifera* flower development based on the same samples used for metabolome analysis. A total of 1,163,327,390 clean reads representing a total of 174.49 Gb nucleotides were generated, with an average GC content of 48.03% (Supplementary Table 7). After de novo transcriptome assembly, a total of 224,245 unigenes were obtained, with an N50 length of 2421 bp (Supplementary Table 8). Based on transcriptome annotation and functional classification, 63.22% (141,781) unigenes were assigned putative functional annotations (Supplementary Fig. 9). The five species showing the top BLASTx hits in the NCBI nonredundant protein sequence database were *Nelumbo nucifera* (35.0%), *Vitis vinifera* (14.3%), *Phoenix dactylifera* (7.4%), *Elaeis guineensis* (7.3%), and *Amborella trichopoda* (3.3%, Supplementary Fig. 10). A gene was retained in the subsequent analysis if it was expressed at a sufficient level, with a counts-per-million (CPM) value greater than one in at least two libraries, resulting in 65,184 expressed unigenes. Among these expressed unigenes, 22,096 unigenes were differentially expressed (false discovery rate (FDR) < 0.05) between at least two different samples (Fig. 1). The hierarchal clustering of the significant changes in unigene expression across all samples revealed a stage/tissue-specific transcriptome profile during *L. tulipifera* flower development (Fig. 1).

The *Liriodendron* lineage belongs to the Magnoliaceae family, which resides within the magnoliids, representing an early-diverging lineage of Mesangiospermae. Thus, this group offers unique insight into early angiosperm evolution, especially flower evolution. We identified 91 MADS-box unigenes, which could be classified into two types, type I (Mα, Mβ, and Mγ) and type II (MIKCC and MIKC*), based on phylogenetic analyses (Supplementary Figs. 11 and 12). These MADS-box unigenes included homologous genes involved in the classic ABCE model of floral organ identity. Specifically, AP1/FUL and AGL6 exhibit A function activity for sepal and petal identities, AP3 and PI present B-function activity for petal and stamen identities, and AG shows C function activity for stamen and carpel identities. In addition, the E-function protein SEP1 is required for interacting with ABC-function proteins. Consistent with the ABCE model, we found that two *AGL6* homologs (*Cluster-30529.89360* and *Cluster-30529.93650*) were both highly expressed in petals and sepals, while most *PI* homologs exhibited a higher expression level in petals than in sepals during tuliptree flower development (Supplementary Fig. 13). However, homologs of another B-function gene, *AP3*, presented no obvious bias between the petals and sepals (Supplementary Fig. 13b). Moreover, we found one SEP homolog (*Cluster-30529.29397*) that was highly expressed in petals and almost absent in sepals, while the remaining homologs were expressed in both petals and sepals (Supplementary Fig. 13d). These results suggested that flower development may not strictly comply with the ABCE model, at least regarding petal/sepal differentiation, in *Liriodendron*.

### Time-course RNA-seq analysis during petal coloration

**Fig. 2:**
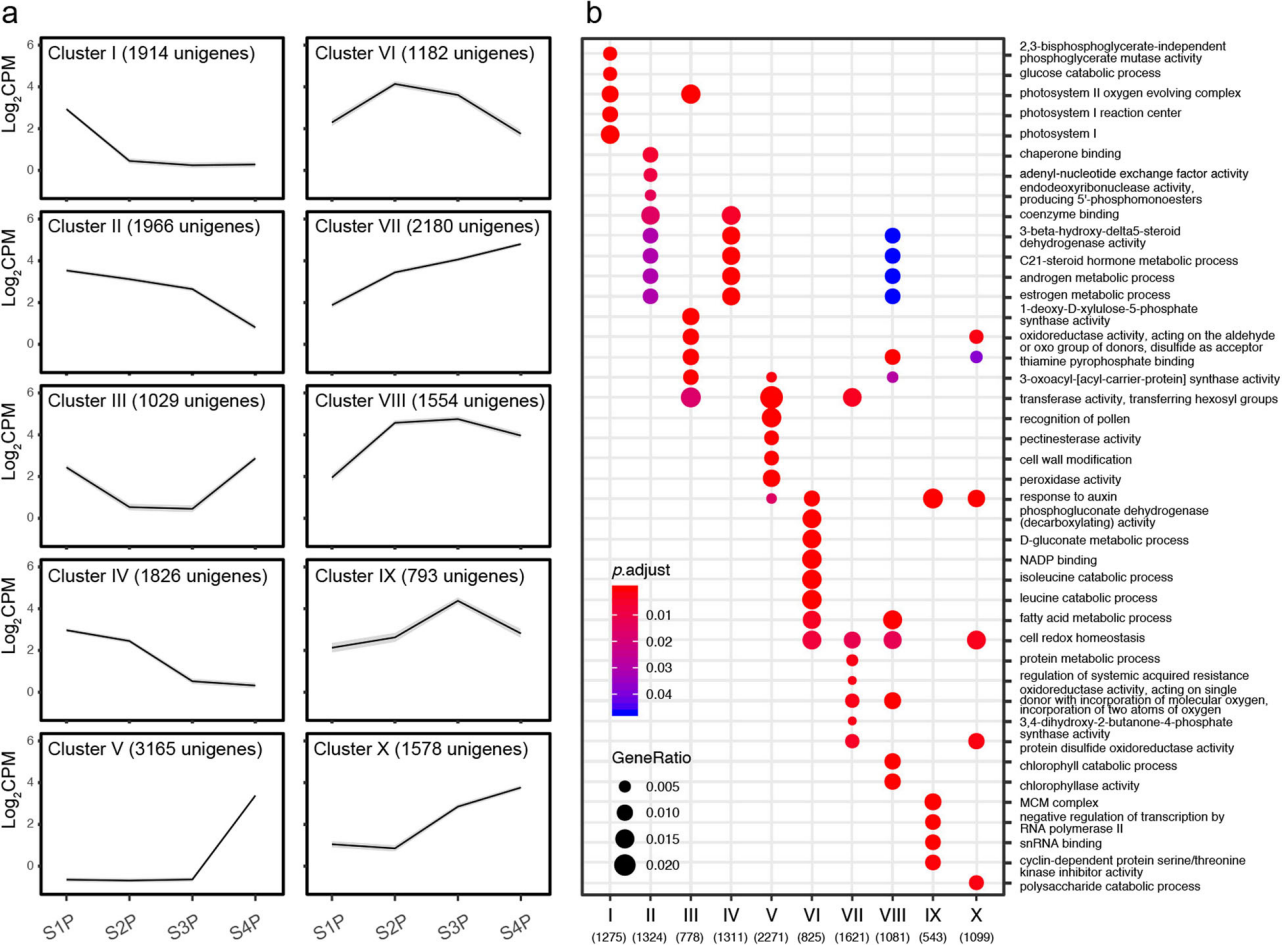
Transcript abundance of differentially expressed genes (DEGs) during *Liriodendron* petal development. **a** *K*-means clustering analysis of the DEGs into ten clusters according to their expression profile. The cluster names and the number of unigenes for each cluster are indicated. **b** Comparison of the GO enrichment of unigene clusters. The sizes of the dots represent the percentage of each row (GO annotation), and *p* values were calculated from hypergeometric tests

We performed an analysis of variance (ANOVA)-like test for any expression differences among all four stages, resulting in 17,187 unigenes that displayed dynamic expression patterns throughout *L. tulipifera* petal development. To determine clusters in which genes shared the same expression pattern, we used the *K*-means clustering algorithm, which grouped genes based on the similarity of their transcriptome profiles. A total of 10 clusters were identified (Fig. 2a), which could be roughly classified into four types: upregulated (clusters V, VII, and X), downregulated (clusters I, II, and IV), up- then downregulated (clusters VI, VIII, and IX), and down- then upregulated (cluster III). Among the three clusters in which gene expression exhibited a general downward trend, cluster I consisted of genes that were strongly expressed at the earliest stage and then rapidly decreased to a very low expression level from the second stage onward. Gene ontology (GO) enrichment analysis showed that three photosynthesis-related terms, “photosystem I,” “photosystem I reaction center,” and “photosystem II oxygen evolving complex,” were enriched in this cluster (Fig. 2b). Considering these results in combination with the results of morphological identification (Supplementary Figs. 1 and 2), we proposed that *L. tulipifera* petals showed reduced photosynthetic activity as soon as petal band coloration began. Contrary to the expression pattern of cluster I, cluster V consisted of genes that were barely expressed at the first three stages, after which their expression rapidly increased to a very high level at the last stage. Some GO terms related to pollen, such as “recognition of pollen” and “pectinesterase activity”^27^, and to senescence, such as “peroxidase activity”^28^, were enriched in this cluster. Since *L. tulipifera* petals remain green with high photosynthetic activity at the first stage, start to show coloration at the second stage, and become senescent at the last stage, we focus more on the genes that were more highly expressed at the two middle stages, especially since the second stage, that is, clusters VI and VIII. However, the only two common GO terms that were overrepresented in clusters VI and VIII were “fatty acid metabolic process” and “cell redox homeostasis”.

### Comparative transcriptome analyses

**Fig. 3:**
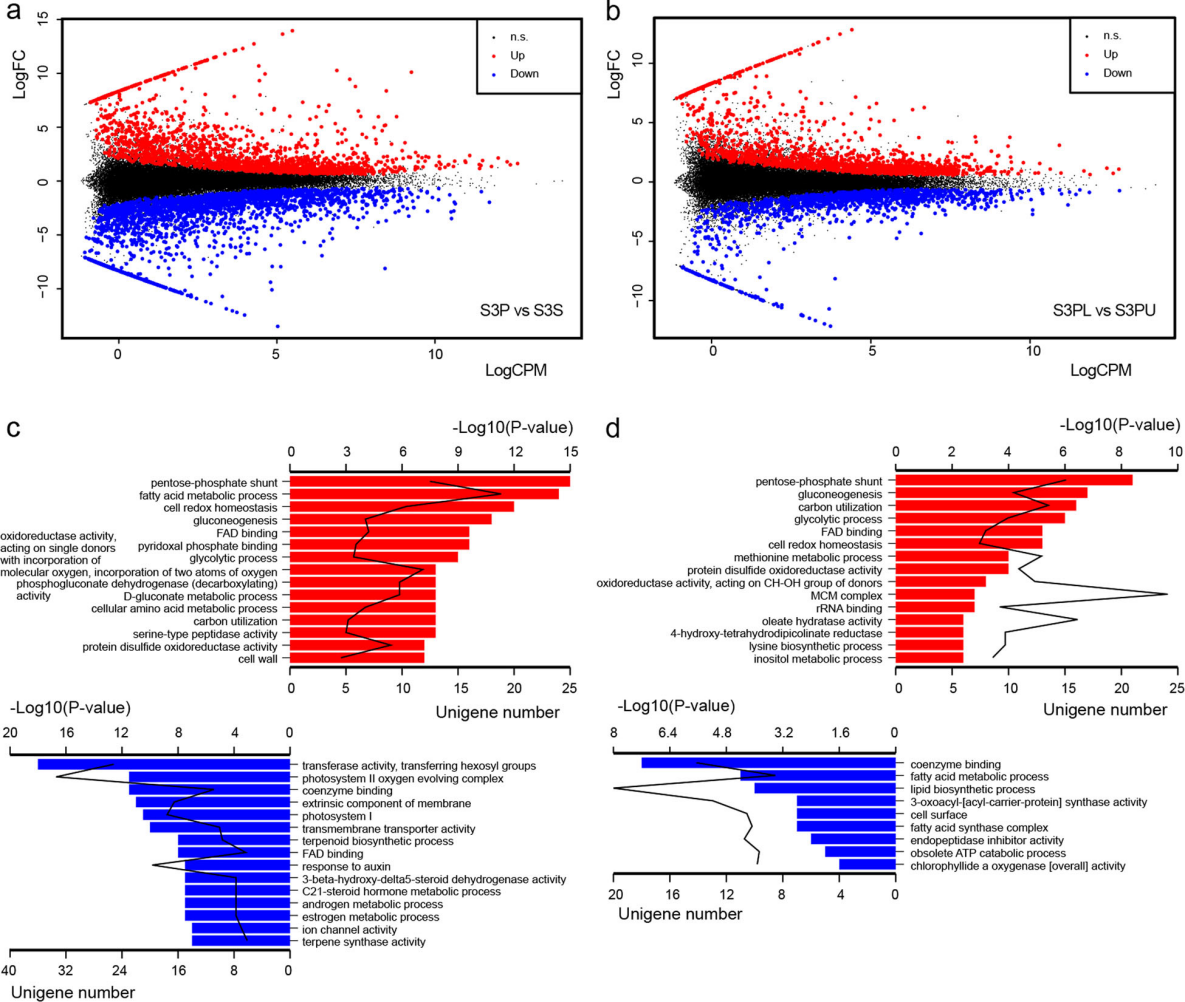
Comparative analyses of the *Liriodendron* petal transcriptomes. **a** Comparative transcriptome analysis of petals and sepals at the third developmental stage, as defined in Supplementary Fig. 1. **b** Comparative transcriptome analysis of the lower and upper sides of the petals at the third development stage. The log-fold change (LogFC) for each unigene is plotted against the log-counts-per-millions (LogCPM). Significantly differentially expressed unigenes at an FDR of 5% are highlighted in red for upregulation and green for downregulation. **c** GO enrichment of unigenes that are differentially expressed between petals and sepals. **d** GO enrichment of unigenes that are differentially expressed between the lower and upper sides of petals. The bar graph represents the unigene number, and the line graph represents the −log 10(*p* value)

To narrow down the list of candidate genes for *L. tulipifera* petal coloration, especially petal band coloration, we also performed two comparative transcriptome analyses. One consisted of a comparison between petals and sepals, resulting in 1866 upregulated and 2200 downregulated unigenes in petals compared to sepals (Fig. 3a). The other consisted of the comparison of the lower versus upper sides of petals, resulting in 1549 upregulated and 1279 downregulated unigenes (Fig. 3b). Compared with petals, the genes that presented a higher expression in sepals were enriched in two GO terms related to photosynthesis, “photosystem II oxygen evolving complex” and “photosystem I” (Fig. 3c). This result was consistent with the observed phenomenon of the sepals of *L. tulipifera* always remaining green (Supplementary Fig. 1). Notably, the GO term “fatty acid metabolic process” was overrepresented among the unigenes that were upregulated in petals compared to sepals (Fig. 3c) and the unigenes that were upregulated on the upper side of petals compared to the lower side of petals (Fig. 3d). These results suggested that fatty acids might play an important role during petal development, but not in petal band coloration in *L. tulipifera*.

### Carotenoid biosynthetic pathways during petal coloration

**Fig. 4:**
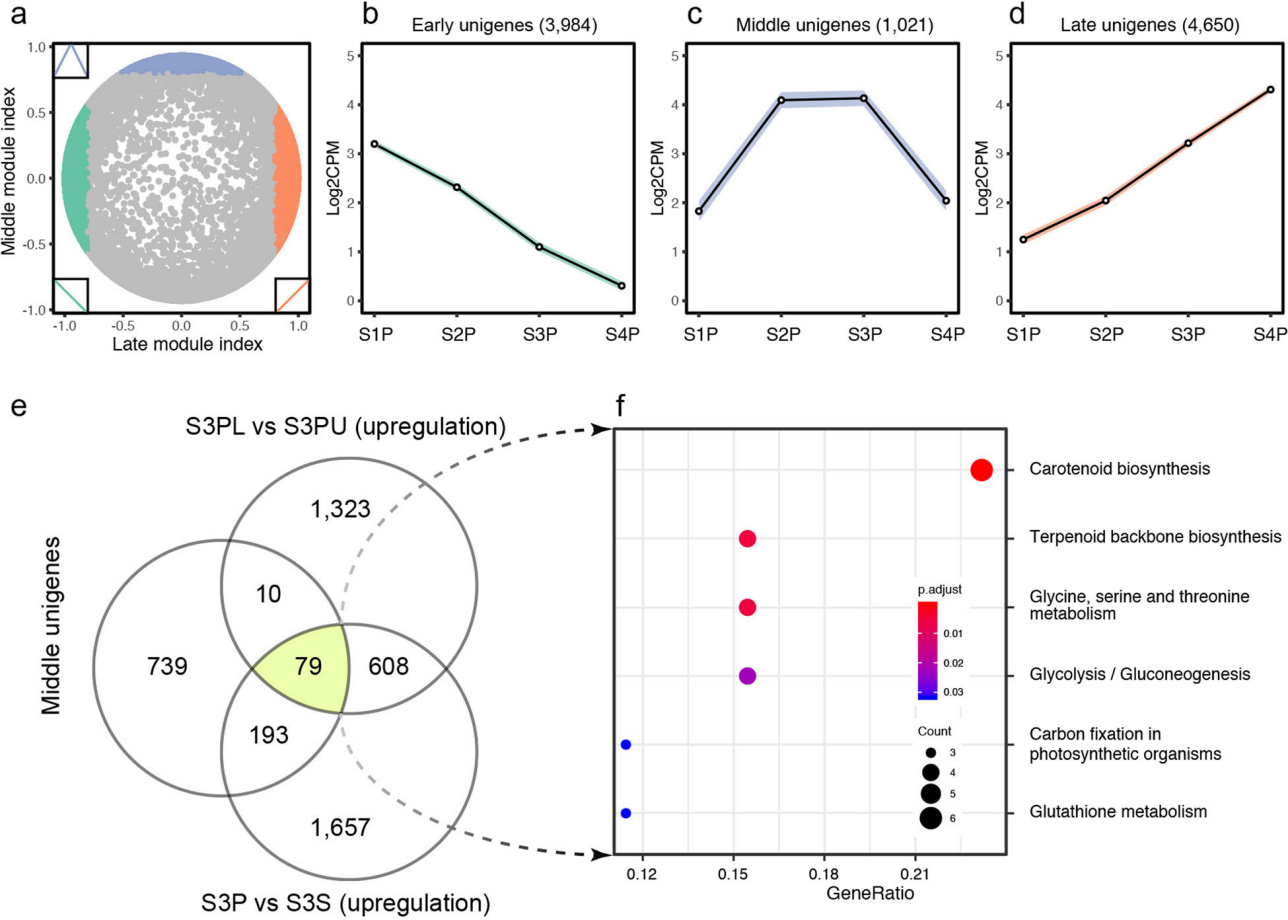
Integration analysis of three independent transcriptome datasets. **a** A landscape showing the correlation between gene expression and two idealized modules, that is, the late module (*x*-axis) and the middle module (*y*-axis). The idealized module profiles are shown in the insets. Spots correspond to unigenes and are colored according to their expression pattern; that is, early unigenes are colored green (**b**), middle unigenes are colored blue (**c**), and late unigenes are colored orange (**d**). **e** The Venn diagram of three datasets, including one time-series dataset and two comparative datasets. **f** KEGG enrichment of unigenes that are commonly shared in all three datasets. The sizes of the dots represent the number of unigenes included in each row (KEGG pathway), and *p* values were calculated from hypergeometric tests

**Fig. 5:**
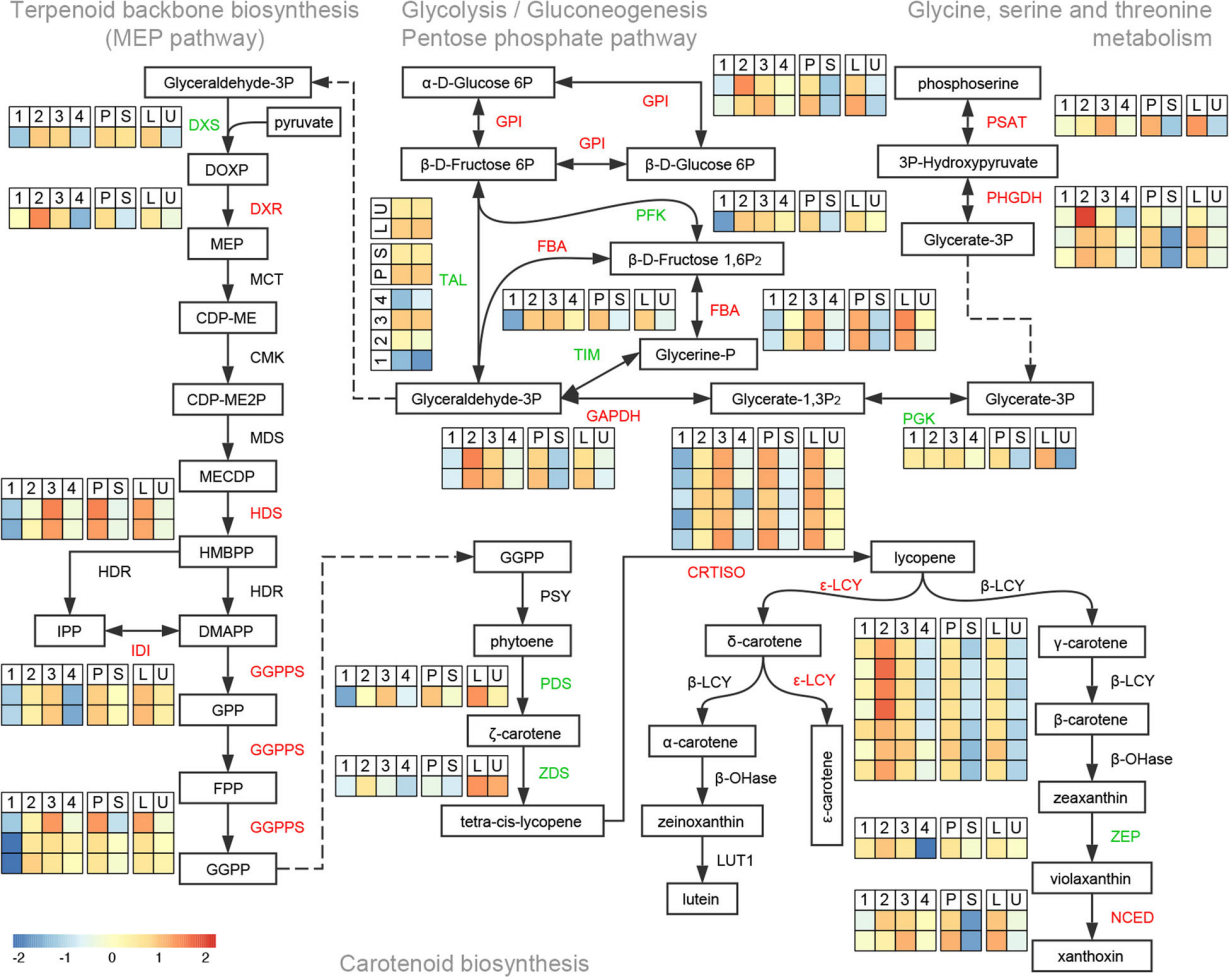
Genes involved in coloration in the petal development of *L. tulipifera*. Relative expression profiles (blue-yellow-red scale) of unigenes implicated in petal coloration. Unigenes with a middle expression pattern in the time-series dataset are shown in red if they are significantly upregulated in both P and L compared with S and U, respectively, and green if they are upregulated, but nonsignificantly so, in either or both of these comparative datasets. Detailed gene names, annotations, and mRNA-seq expression data are provided in Supplementary Fig. 1. 1, S1P; 2, S2P; 3, S3P; 4, S4P; P, S3P; S, S3S; L, S3PL; U, S3PU; PSAT, phosphoserine aminotransferase; PHGDH, d-3-phosphoglycerate dehydrogenase; PGK, phosphoglycerate kinase; GAPDH, glyceraldehyde 3-phosphate dehydrogenase; GPI, glucose-6-phosphate isomerase; PFK, phosphofructokinase; FBA, fructose-bisphosphate aldolase; TIM, triosephosphate isomerase; TAL, transaldolase; DXS, 1-deoxy-d-xylulose-5-phosphate synthase; DOXP, 1-deoxy-d-xylulose 5-phosphate; DXR, 1-deoxy-d-xylulose-5-phosphate reductoisomerase; MEP, 2-C-methyl-d-erythritol 4-phosphate; MCT, 2-C-methyl-d-erythritol 4-phosphate cytidylyltransferase; CDP-ME, 4-(cytidine 5′-diphospho)-2-C-methyl-d-erythritol; CMK, 4-(cytidine 5′-diphospho)-2-C-methyl-d-erythritol kinase; CDP-ME2P, 4-(cytidine 5′-diphospho)-2-C-methyl-d-erythritol 2-phosphate; MDS, 2-C-methyl-d-erythritol 2,4-cyclodiphosphate synthase; MECDP, 2-C-methyl-d-erythritol 2,4-cyclodiphosphate; HDS, (*E*)-4-hydroxy-3-methylbut-2-enyl diphosphate synthase; HMBPP, 1-hydroxy-2-methyl-2-butenyl 4-diphosphate; HDR, (*E*)-4-hydroxy-3-methylbut-2-enyl diphosphate reductase; IPP, isopentenyl diphosphate; DMAPP, dimethylallyl diphosphate; IDI, isopentenyl diphosphate isomerase; GGPPS, geranylgeranyl diphosphate synthase; GPP, geranyl diphosphate; FPP, farnesyl diphosphate; GGPP, geranylgeranyl diphosphate; *PSY*, phytoene synthase; *PDS*, phytoene desaturase; *ZDS*, ζ-carotene desaturase; *CRTISO*, carotenoid isomerase; *ε-LCY*, lycopene ε-cyclase; *β-LCY*, lycopene β-cyclase; β-OHase, β-carotene hydroxylase; LUT1, carotene ε-monooxygenase; ZEP, zeaxanthin epoxidase; NCED, 9-*cis*-epoxycarotenoid dioxygenase. Adapted from Cazzonelli et al.^30^, Nisar et al.^53^, and KEGG (https://www.kegg.jp/)

We referred to genes that exhibited relatively low expression at the first and last stages and relatively high expression at the two middle stages as “middle genes”. Considering that there were two clusters (clusters VI and VIII) that might fit the expression pattern of middle genes (Fig. 2a), we performed a new method to determine all middle genes from the time-course transcriptome profile^29^, resulting in 1021 unigenes (Fig. 4a–d). There were only 79 unigenes that overlapped among the three transcriptome datasets: 1021 middle genes from the time-course dataset, 1866 genes upregulated in petals versus sepals, and 1,549 genes upregulated on the lower side versus the upper side of petals (Fig. 4c). KEGG enrichment analysis showed that only six pathways were significantly overrepresented among these 79 unigenes (Fig. 4d). Furthermore, the first four pathways, comprising one amino acid metabolism pathway (i.e., glycine, serine, and threonine metabolism), one carbohydrate metabolism pathway (i.e., glycolysis/gluconeogenesis), and two terpenoid and polyketide metabolism pathways (i.e., terpenoid backbone biosynthesis and carotenoid biosynthesis), can be integrated into a cascade metabolic network (Fig. 5). Basically, d-glycerate 3-phosphate (glycerate-3P) produced from the “glycine, serine, and threonine metabolism” pathway is used by the “glycolysis/gluconeogenesis” pathway to produce the glyceraldehyde-3P substrate for “terpenoid backbone biosynthesis.” Then, the methylerythritol 4-phosphate (MEP) pathway produces geranylgeranyl diphosphate (GGPP) as the substrate for the “carotenoid biosynthesis” pathway, which produces all kinds of carotenoids, which represent one of the three major plant pigment categories.

We mapped all unigenes that fit the pattern of middle genes during the four petal development stages and the genes that were upregulated in petals compared to sepals and on the lower side of petals compared to the upper side of petals to this network (Fig. 5). Notably, three unigenes could be mapped to GGPP synthase (GGPPS), which catalyzes the biosynthesis of GGPP (Supplementary Table 9). In addition, for each of two genes encoding rate-limiting enzymes (*CRTISO* and *ε-LCY*^30^) in the carotenoid biosynthesis pathway, more than five unigenes that fit the pattern were mapped (Supplementary Table 9). These results suggested that in *L. tulipifera* petals, the local transcriptional regulation of gene expression might lead to the biosynthesis and accumulation of carotenoids for the coloration of the petal band during flower development.

### Metabolomics of carotenoids

To further confirm the role of carotenoids in petal band coloration in *L. tulipifera*, we conducted a targeted metabolomics experiment using the same samples employed for the nontargeted metabolome and transcriptome analyses. We detected the metabolic content dynamics of 18 compounds from the carotenoid biosynthesis pathway during *L. tulipifera* flower development, among which seven compounds were excluded due to too many missing values (Supplementary Table 10). Among the remaining 11 compounds, three xanthophylls (lutein, neoxanthin, and violaxanthin), all of which are downstream products in the carotenoid biosynthesis pathway, decreased gradually during petal development and were upregulated in sepals and the upper side of petals compared to petals and the lower side of petals, respectively (Supplementary Fig. 14). Another xanthophyll, zeaxanthin, which is derived from β-carotene and transformed into violaxanthin, remained at a relatively low level in the first three stages and dramatically increased to a relatively high level in the last stage. In addition, the sepals accumulated much higher levels of zeaxanthins at the third stage than the petals. These results suggested that these xanthophylls may not be the key pigments in the coloration of the petal band during *L. tulipifera* flower development.

Among the carotenes, β-carotene remained stable across different stages and tissues, and α-carotene generally increased during petal development, yet it accumulated at much higher levels in sepals than in petals (Supplementary Fig. 14). The remaining two carotenes, γ-carotene and ε-carotene, increased during petal development and were upregulated in petals and on the lower side of petals compared to sepals and the upper side of petals, respectively (Supplementary Fig. 14). Notably, the absolute content of γ-carotene was somewhat higher than that of ε-carotene, and γ-carotene was enriched on the lower side of petals, while it was almost absent from the upper sides of petals. Since most of the petal surface of interspecific hybrids between the two *Liriodendron* species is colored by orange pigments, in contrast to the narrow band of pigmentation observed in *L. tulipifera* petals^20^, we detected the absolute contents of these four carotenes in the petals of two *Liriodendron* hybrids (*L. chinense* × *L. tulipifera*), LH#1 and LH#4, in which the orange band extends across almost the whole petal (Supplementary Fig. 15a). Compared with the other carotenes, the petals of the *Liriodendron* hybrids exhibited an extremely high content of γ-carotene (Supplementary Fig. 15b). Taken together, the site-specific local accumulation of γ-carotene may be the key process that contributes to petal band coloration during *L. tulipifera* flower development.

### Key enzymes for carotene dynamics

To determine the key enzymes involved in the local accumulation of pigments in the petal band of *L. tulipifera*, we explored the expression of all genes involved in the biosynthesis and catabolism of carotenes (Supplementary Fig. 16). Strikingly, the genes involved in the conversion of GGPP to lycopene included at least one unigene that was highly expressed across different tissues (petals and sepals) and parts of petals (lower and upper sides), such as *PSY* (*phytoene synthase*), *PDS* (*phytoene desaturase*), or *ZDS* (*ζ-carotene desaturase*). For *CRTISO*, *Cluster-30529.109281* belonged to the middle genes based on expression pattern clustering (Fig. 4) and was upregulated in petals and on the lower side of petals compared to sepals and the upper side of petals, respectively (Fig. 3). However, the expression of *Cluster-30529.109281* was relatively high in sepals (a logCPM value of 6.02) and on the upper side of petals (a logCPM value of 7.17), suggesting that the biosynthesis of lycopene was not the key factor determining the petal coloration pattern in *L. tulipifera*.

Subsequently, lycopene is transformed into γ-carotene by β-lycopene cyclase (*β-LCY*) or δ-carotene by *ε-LCY*. Next, γ-carotene can be further transformed into β-carotene by *β-LCY*, and δ-carotene can be further transformed into ε-carotene by *ε-LCY* or α-carotene by *β-LCY*. Interestingly, we detected only one unigene (*Cluster-30529.139776*) that encoded *β-LCY*, which presented relatively stable expression across different tissues and parts of petals (Supplementary Fig. 16). In contrast, we detected 14 expressed unigenes encoding *ε-LCY*, most of which exhibited relatively high expression at the two middle stages and were upregulated in petals and on the lower side of petals compared to sepals and the upper side of petals, respectively. More importantly, all of these *ε-LCY*-encoding unigenes were expressed at a relatively low level in sepals or on the upper side of petals (Supplementary Fig. 16). In addition, genes involved in the conversion of carotenes to downstream metabolites, such as LUT5 (β-ring hydroxylase), LUT1 (carotene ε-monooxygenase), and β-OHase (β-carotene hydroxylase), seemed to have little to do with the petal coloration pattern of *L. tulipifera* at the transcriptional level since they were expressed stably across different tissues and parts of petals (Supplementary Fig. 16).

There are currently only two magnoliid genomes available^31^ for *L. chinense*^32^ and *Cinnamomum kanehirae*^33^. Both of these genomes contain only one *ε-LCY* gene and one *β-LCY* gene. We reconstructed the phylogenetic tree of *LCY* genes using *capsanthin/capsorubin synthase* (*CCS*) genes as the outgroup based on four eudicots, four monocots, three magnoliids, one basal angiosperm, and one lycophyte (Supplementary Fig. 17). The *ε-LCY* and *β-LCY* genes were obviously grouped into two distinct clades, with all *CCS* genes being grouped together. Within the *β-LCY* clade, only one *β-LCY* gene came from *L. chinense*, which was clustered with *Cluster-30529.139776* as a sister group, and the *β-LCY* gene of *C. kanehirae* constituted the next group. Interestingly, within the *ε-LCY* clade, all unigenes from *L. tulipifera* clustered together with the only *ε-LCY* gene of *L. chinense*, with *Cluster-30529.146811* being the most closely related, while the others clustered into several subgroups, suggesting that there was likely more than one *ε-LCY* gene copy in the *L. tulipifera* genome. Consistent with this, we identified two additional copies of *ε-LCY* in the *L. tulipifera* genome (Supplementary Table 11; unpublished data). Among the unigenes that were de novo assembled from the LH#1 and LH#4 petal transcriptomes (Supplementary Table 11; unpublished data), the unigene that clustered with the *L. tulipifera ε-LCY* genes presented the highest expression (Supplementary Fig. 18), which might be related to the extremely high content of γ-carotene in the petals of these two *Liriodendron* hybrids (Supplementary Fig. 15).

In addition, we performed quantitative real-time PCR (qRT-PCR) experiments to determine the expression of 11 carotenoid biosynthesis genes within the petal band area, and one *ε-LCY* unigene (*Cluster-30529.103712*) was found to be highly upregulated in the colored petal band, while the remaining genes were relatively stable across different petal developmental stages (Supplementary Fig. 19). Moreover, we also quantified the expression of only one *ε-LCY* gene (*Lchi18362*) in *L. chinense* within the petal area corresponding to the petal band of *L. tulipifera*, showing a relatively stable expression pattern across different petal developmental stages (Supplementary Fig. 19).

### Potential transcriptional regulation mechanisms

**Fig. 6:**
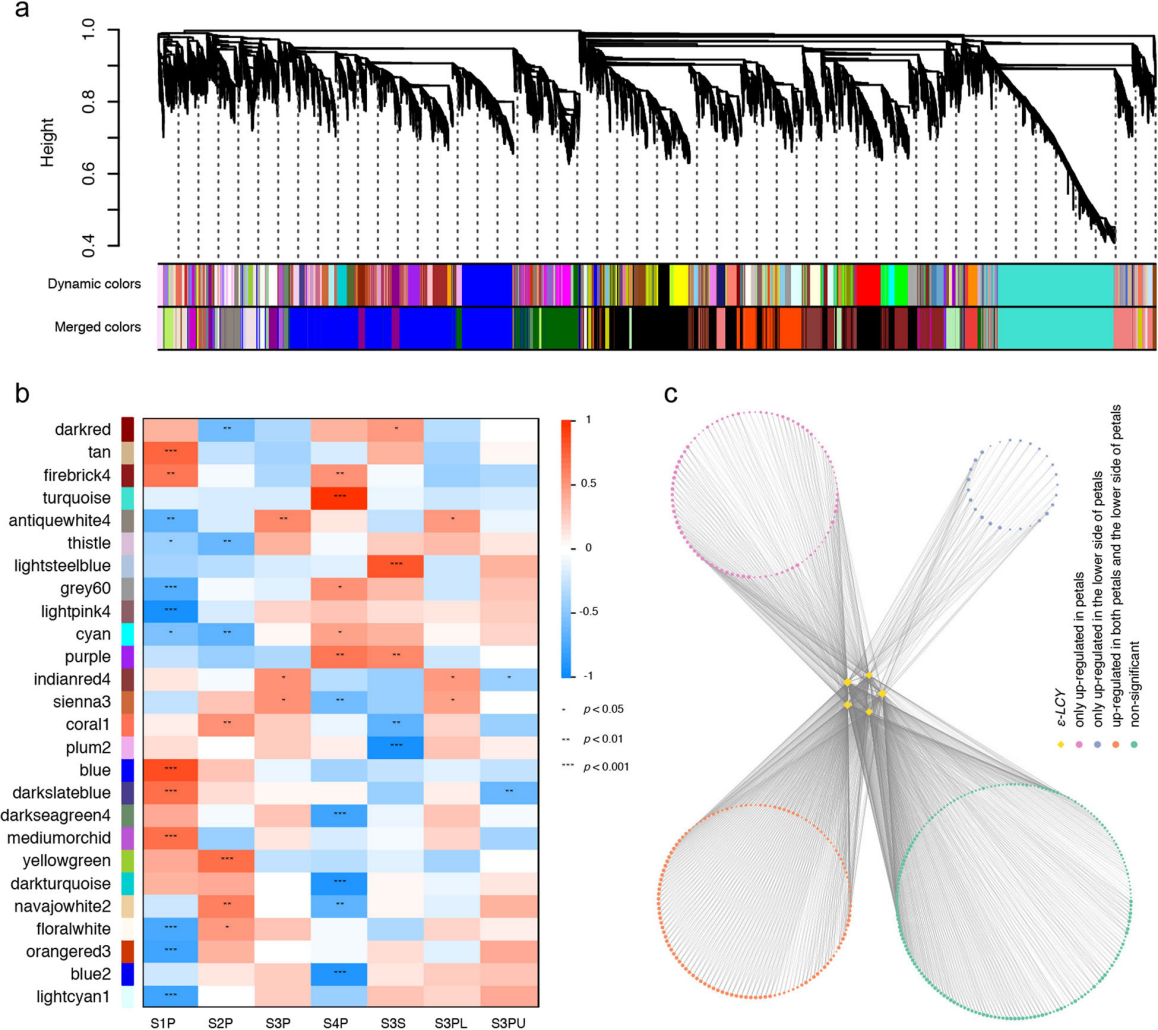
Construction of the gene coexpression network during *L. tulipifera* flower coloration through WGCNA. **a** Gene dendrogram obtained by hierarchical clustering with the module color indicated by the color of the row underneath. A total of 26 distinct modules were identified. **b** Relationships of modules and different samples including petals in four developmental stages (S1P, S2P, S3P, and S4P), sepals (S3S), and the lower (S3PL) and upper (S3PU) sides of petals at the third stage. Each row in the table corresponds to a module, and each column corresponds to a sample. **c** Unigenes whose expression is highly correlated with five *ε-LCY* unigenes in the module coral1

To identify potential molecular mechanisms underlying this local transcriptional regulation of carotene accumulation in *L. tulipifera*, we performed weighted gene coexpression network analysis (WGCNA) to identify modules in which genes were highly coexpressed. We obtained a total of 26 distinct modules, among which four modules (i.e., coral1, darkturquoise, lightcyan1, and sienna3) were enriched in the KEGG pathway “carotenoid biosynthesis” (Fig. 6a and Supplementary Fig. 20). However, both darkturquoise and lightcyan1 modules exhibited a stronger positive relationship with the upper side than the lower side of petals (Fig. 6b). Nine of the 14 expressed *ε-LCY* unigenes were included in coral1, five of which were significantly upregulated in sepals and on the lower side of petals compared to petals and the upper side of petals, respectively (Supplementary Fig. 21), while only one was included in sienna3, and none were included in the remaining two modules. There were 429 unigenes coexpressed with these five *ε-LCY* unigenes, among which 217 unigenes were upregulated in petals compared to sepals, and 152 unigenes were upregulated on the lower side compared to the upper side of petals, with an overlap value of 125 (Fig. 6c). Among these 125 unigenes, we found a bHLH TF (*Cluster-30529.26678*) with gene expression that was highly correlated with these five *ε-LCY* unigenes and showed no or barely detectable expression in sepals and on the upper side of petals (Supplementary Fig. 22 and Supplementary Table 12).

To further determine the key gene set for petal band coloration in *L. tulipifera*, we filtered all unigenes with a specific expression pattern, including (1) an upward–downward trend during petal development, (2) upregulation both in petals and on the lower side of petals, and (3) no or barely detectable expression (logCPM <0) in both sepals and on the upper side of petals. Finally, we obtained a total of 49 unigenes that fit these criteria well (Supplementary Table 13). The gene expression correlation analysis showed that there were two groups exhibiting differences in expression at the first stage during petal development (Supplementary Fig. 23). The bHLH TF *bHLH96* (*Cluster-30529.26678*) and two *ε-LCY* genes (*Cluster-30529.109365* and *Cluster-30529.153215*) were classified into the first group, which was characterized by being expressed at the first stage. In contrast, the unigenes classified into the second group presented barely detectable or relatively low expression at the first stage, including two *CRTISO* genes (*Cluster-30529.138317* and *Cluster-30529.163758*) and one *ε-LCY* gene (*Cluster-30529.131959*). Interestingly, this group also contained one unigene, *Cluster-30529.168715*, encoding the ER-localized PIN5 auxin transporter^34^.

## Discussion

### Preband formation precedes petal pigmentation

At the first stage, the band area in which orange pigments will accumulate gradually during the development of petals already seemed to be clearly defined and showed an obvious pale color, whereas the rest of the petal area was green (Supplementary Fig. 1). In plants, green coloration is mainly contributed by chlorophylls, a class of Mg^2+^-containing tetrapyrrole compounds. Thus, we speculated that an early step in petal band development involved the local repression of chlorophyll biosynthesis and/or the activation of chlorophyll degradation. Consistent with this, we found that genes that were downregulated on the lower side of petals were significantly enriched for the GO term of “chlorophyllide a oxygenase activity,” which is associated with chlorophyll *zz* biosynthesis^35^ (Fig. 3d). Furthermore, we found that two chlorophyll biosynthesis*-*related genes, *Cluster-30529.88746*, encoding glutamyl-tRNA reductase^36^, and *Cluster-30529.75818*, encoding Mg-protoporphyrin IX monomethylester cyclase^37^, were downregulated on the lower side compared to the upper side of petals. In addition, we also found that a unigene (*Cluster-30529.87029*) encoding a cytokinin dehydrogenase (CKX) that is responsible for irreversible cytokinin degradation in plants^38^ was upregulated with a logFC value of 11 on the lower side of petals compared to the upper side of petals. Since cytokinin signaling can positively regulate chlorophyll biosynthesis and chloroplast biogenesis^39^, we speculated that the local activation of the *CKX* gene may also contribute to the loss of green pigmentation in the band area of *L. tulipifera* petals. Although all these results were observed at the third stage in petals, the local repression of chlorophyll biosynthesis was at least required to maintain the loss of green pigmentation from the band area during the petal development of *L. tulipifera*. Additionally, genes that were specifically and highly expressed at the first stage were significantly enriched only for photosynthesis-related terms (Fig. 2). On the basis of the phenotypic identification of developing petals, gene profiling comparisons between band and non-band areas and functional enrichment analysis among genes with different patterns, we inferred that the band area was already well defined prior to band pigmentation, partially by locally repressing chlorophyll biosynthesis, which is also important for band identity maintenance during petal development in *L. tulipifera*.

### Carotenoid biosynthesis contributes to *L. tulipifera* petal band coloration

Beginning in the second stage during petal development, the band area started to show coloration with orange pigments (Fig. 1). By combining transcriptome and metabolome profiling studies, we found one main pathway cascade that contributed to the band-specific pigmentation (Fig. 4). This pathway cascade starts with “glycine, serine, and threonine metabolism,” to produce glycerate-3P, which is then converted into glyceraldehyde-3P via the “glycolysis/gluconeogenesis” pathway; subsequently, glyceraldehyde-3P is converted into GGPP as the substrate for the “carotenoid biosynthesis” pathway via one of the upstream pathways of terpenoid backbone biosynthesis (i.e., the MEP pathway) (Fig. 5). The whole pathway cascade is specifically transcriptionally activated in the band area during petal development, with two rate-limiting enzymes, CRTISO and ε-LCY, being especially notable for the expression of their encoding unigenes (Fig. 5). However, the phenotype identification and carotenoid metabolomic analyses of both *L. tulipifera* and *Liriodendron* hybrid petals suggested that γ-carotene, a rare carotene in plants^40^, was mainly responsible for the orange pigmentation of *Liriodendron* petals (Supplementary Fig. 15). As we all know, γ-carotene is an intermediate compound produced from lycopene by *β-LCY* that is converted into β-carotene by the same enzyme (Supplementary Fig. 16). We only detected one unigene (*Cluster-30529.139776*) encoding *β-LCY* showing no obvious expression dynamics (Supplementary Fig. 16), consistent with the dynamics of β-carotene across all samples (Supplementary Fig. 14). The following phylogenetic analysis indicated that there was likely more than one *ε-LCY* copy in *L. tulipifera*, which was further confirmed by gene prediction according to the *L. tulipifera* Genome Project, whereas only one copy was predicted in the *L. chinense* genome (Supplementary Fig. 17 and Supplementary Table 11), possibly partially explaining the difference in petal coloration between this pair of sister species due to some as-yet-unknown mechanism. One explanation is that *β-LCY* exhibits higher activity in the conversion of lycopene into γ-carotene than that of γ-carotene into β-carotene, leading to the petal band-specific accumulation of γ-carotene. However, this cannot explain the γ-carotene dynamics across petal developmental stages. Additionally, an alternative explanation for the petal band coloration of *L. tulipifera* is that the protein encoded by this additional *ε-LCY* copy might have evolved a novel catalytic activity that can convert lycopene or other carotenes to γ-carotene, but not γ-carotene to β-carotene, ultimately leading to the petal band-specific accumulation of γ-carotene during tuliptree flower development. Future studies should examine which *LCY* genes actually function in the determination of petal band coloration in *L. tulipifera*, whether an additional *ε-LCY* copy truly exists in *L. tulipifera* and, if so, whether the encoded protein associates with the petal band-specific accumulation of γ-carotene.

### Underlying mechanisms of band-specific pigmentation

*Liriodendron tulipifera* petal band coloration involves two processes: the loss of green pigmentation and orange pigment accumulation. We found that many chlorophyll biosynthesis genes were downregulated in the band area during petal development. We also identified one cytokinin degradation gene that was upregulated in the band area, which may contribute to the loss of green pigmentation by repressing cytokinin signaling. Previous studies showed that two TFs, HY5 and GOLDEN2-LIKE2 (GLK2), function downstream of light and cytokinin signaling to coordinate the expression of key genes involved in chloroplast biogenesis in *Arabidopsis*^39^. Consistent with this, we found that two unigenes, *Cluster-30529.58264* and *Cluster-30529.67286*, encoding the HY5 TF, were both downregulated, although not significantly so, on the lower side of petals. Two other unigenes, *Cluster-30529.78854* and *Cluster-30529.78852*, encoding the GLK2 TF, were both significantly downregulated on the lower side of petals. Thus, we speculated that these two TFs may be both important for band area formation and maintenance by integrating light and cytokinin signaling to promote the band-specific loss of green pigmentation during petal development in *L. tulipifera*.

In terms of orange pigment accumulation, we identified a pathway cascade (ultimately leading to carotenoid biosynthesis) that was specifically activated in the band area during petal development in *L. tulipifera* (Fig. 5). A number of TFs with a regulatory function in carotenoid biosynthesis belong to the R2R3-MYB protein family (Supplementary Table 1); thus, we identified 386 unigenes possessing a Myb-like DNA-binding domain (PF00249), among which only *Cluster-30529.96691* was significantly upregulated both in petals and on the lower side of petals compared to sepals and the upper side of petals, respectively (Supplementary Table 14). The most closely related gene to *Cluster-30529.96691* in *Arabidopsis* is *MYB-RELATED PROTEIN 1* (*MYR1*), which acts redundantly with *MYR2* to repress flowering and organ elongation, partly by decreasing *GA20ox2* expression and the bioactive GA_4_ content^41^. Similar to *MYR1*, the expression of a *MYR2* homolog in *L. tulipifera* (*Cluster-30529.146042*) was upregulated, but not significantly so, both in petals and on the lower side of petals compared to sepals and the upper side of petals, respectively (Supplementary Table 14). However, no *GA20ox2* homolog expression was detected during tuliptree flower development. Notably, we found that two bioactive GA_7_ compounds (Com_1341_neg and Com_1342_neg) were significantly downregulated on the lower side compared to the upper side of petals (Supplementary Table 6). Considering that the band area remained almost unchanged during petal expansion in *L*. tulipifera (Supplementary Fig. 2), we speculated that the repression of bioactive GA_7_ biosynthesis, likely mediated by the MYB-related protein MYR1, might be responsible for the repression of petal band expansion during tuliptree flower development.

To determine the potential regulatory network underlying petal band-specific coloration in *L. tulipifera*, we performed WGCNA to identify modules of highly correlated genes associated with the petal band-specific coloration pattern in *L. tulipifera*. Finally, we found that most *ε-LCY* unigenes were included in the coral1 module, which was highly correlated with the middle stage in the developing petal, especially with the colored part of the petals (Fig. 6). We constructed a network of genes that were highly coexpressed with these *ε-LCY* unigenes. Among these genes, a bHLH TF (*Cluster-30529.26678*) was noted due to showing a high correlation with *ε-LCY* unigenes and barely detectable expression in sepals and on the upper side of petals (Supplementary Fig. 22). Its most closely related gene in *Arabidopsis* is *bHLH96* (*AT1G72210*), which is highly expressed in petals^42^ and stomatal guard cells^43^, but has no available detailed functional information. Considering that the modified stomata are the route of nectar exudation^44^ and that tuliptree floral nectar is specifically distributed in the petal band region^45^, we believe that it will be very interesting to determine whether the bHLH96 protein functions in nectary development and/or petal band-specific coloration in *L. tulipifera*.

## Materials and methods

### Plant materials

A *L. tulipifera* tree (provenance: Ontario, Canada) planted in Longshan (Anji, China) was used in this study. First, we selected six different developmental stages of flowers or flower buds based on external characteristics. Then, four representative developmental stages were determined for subsequent omics analyses based on the color change in the petal band. Petals from these four developmental stages were collected for time-course metabolomic and transcriptomic experiments. In addition, the sepals and the lower and upper sides of petals at the third stage were collected for two comparative metabolomic and transcriptomic experiments. In addition, we collected two petal samples from two *Liriodendron* hybrids (LH#1 and LH#4) that were planted at the same location. These two samples were only used for carotenoid quantification. Each sample consisted of six petals or three sepals from the same flower, and three biological replicates were analyzed in all cases. All samples were immediately frozen in liquid nitrogen and then stored at −80 °C without thawing before RNA or metabolite extraction.

### Metabolomics

For each biological sample, a 100 mg fresh weight sample was first frozen in liquid nitrogen and then ground into powder and extracted with *n*-hexane:acetone:ethanol (2:1:1, v/v/v). The extract was vortexed for 30 s, and then ultrasound-assisted extraction was applied for 20 min at room temperature, followed by centrifugation for 5 min at 12,000 r.p.m. Next, we repeated the steps above and collected the supernatant, which was further evaporated to dryness under a nitrogen gas stream, and the residue was reconstituted in 75% methanol. Finally, the solution was centrifuged, and the supernatant was collected for LC with tandem mass spectrometry (LC-MS/MS) analysis. A mixture containing the same volume of supernatant from each biological sample was used as a quality control (QC) sample to evaluate the system’s stability. In addition, blank samples containing the same solvent that was used for the reconstitution of biological samples were run together with the QC samples each day to remove background contamination.

For nontargeted metabolomic analysis, the sample extracts were analyzed using an LC-ESI-MS/MS (LC electrospray ionization MS/MS) system. The analytical conditions were as follows: HILIC column: Thermo Accucore HILIC column (2.6 µm, 3 mm × 100 mm); temperature of the column: 40 °C; flow rate: 0.3 mL/min; mobile phase A (positive): 0.1% formic acid, 95% acetonitrile, and 10 mM ammonium acetate; mobile phase B (positive): 0.1% formic acid, 50% acetonitrile, and 10 mM ammonium acetate; mobile phase A (negative): 95% acetonitrile and 10 mM ammonium acetate (pH adjusted to 9.0); mobile phase B (negative): 50% acetonitrile and 10 mM ammonium acetate (pH adjusted to 9.0); gradient program: 0–1 min, 98% A and 2% B; 1–17 min, 98% to 50% A and 2% to 50% B; 17–17.5 min, 50% A and 50% B; 17.5–18 min, 50% to 98% A and 50% to 2% B; 18–19 min, 98% A and 2% B. The ESI probe was fixed at level C. The parameters of the mass spectrometer were set as follows: full scan range = 100–1500 (*m*/*z*); spray voltage: 3.2 kV; sheath gas flow rate: 35 arb; aux gas flow rate: 10 arb; capillary temp: 320 °C; polarity: positive and negative. Then, we processed the raw data by using the Thermo Compound Discoverer 3.0 software. First, we performed peak picking with a mass tolerance of 5 p.p.m., intensity tolerance of 30%, signal-to-noise threshold of 3, and minimum peak intensity of 100,000. Then, the merging and grouping of features were performed at a radiation tolerance of 0.2 min and a mass tolerance of 5 p.p.m. The blank was used for background subtraction for the removal of potential noise and contaminants from the LC-MS data. Only peaks showing a 3-fold increase or higher in the biological samples compared with the blank samples were retained. Peak areas were normalized to the corresponding peak areas for the QC samples. The metabolites identified in the processed raw data of mass spectral peaks were searched against the mzCloud database for a matching fragmentation spectrum.

For carotenoid metabolomics, the sample extracts were analyzed using an LC-MS/MS system with the following conditions: HPLC column: YMC C30 (3 µm, 2 mm × 100 mm); solvent system: mobile phase A: acetonitrile:methanol (3:1, v/v) and 0.01% butylated hydroxytoluene (BHT); mobile phase B: methyl *tert*-butyl ether and 0.01% BHT; gradient program: 85:5 (v/v) at 0 min, 75:25 (v/v) at 2 min, 40:60 (v/v) at 2.5 min, 5:95 (v/v) at 3 min, 5:95 (v/v) at 4 min, 85:15 (v/v) at 4.1 min, 85:15 (v/v) at 6 min; flow rate, 0.8 mL/min; temperature: 28 °C; injection volume: 5 μL. The effluent was alternatively connected to a triple quadrupole-linear ion trap (Q TRAP)-MS. The API 6500 Q TRAP LC/MS/MS system, equipped with an APCI Turbo Ion-Spray interface, was operated in positive-ion mode and controlled by the Analyst 1.6.3 software (AB Sciex). The APCI source operation parameters were as follows: ion source: turbo spray; source temperature: 350 °C; curtain gas (CUR): 25.0 psi; collision gas (CAD): medium. The declustering potential (DP) and collision energy (CE) for individual multiple reaction monitoring transitions were determined with further DP and CE optimization. We built a MetWare database based on authentic carotenoid standards (BioBioPha, Kunming, Yunnan, China; Sigma Aldrich, St. Louis, MO, USA) for the qualitative analysis of MS data. Then, for absolute quantification, we prepared the solutions for each carotenoid standard with several different concentrations and obtained the peak area values corresponding to each concentration. Next, we separately constructed standard curves for all authentic carotenoid standards. Thereafter, we calculated the concentration values for all carotenoids using their respective standard curves. Finally, we determined the contents of the targeted carotenoids in different biological samples using the formula B*C/1000/*D*, where *B* represents the concentration value calculated using the standard curve, *C* represents the reconstitution volume, and *D* represents the sample weight.

### Transcriptomics

A total of 1.5 μg RNA per sample was used as the input material for the RNA sample preparations. Sequencing libraries were generated using the NEBNext^®^ Ultra^TM^ RNA Library Prep Kit for Illumina^®^ (NEB, USA) and sequenced on an Illumina HiSeq platform from which paired-end reads (150 bp) were generated. After QC, read filtering and base correction for the raw read data, we performed the de novo assembly of the transcriptome using Trinity version 2.4.0^46^. Gene function was annotated based on the following databases: NCBI nonredundant protein sequences, NCBI nucleotide sequences, Pfam, eukaryotic orthologous groups, Swiss-Prot, KEGG, and GO. Then, we used the clean read data to quantify representative gene model expression using Salmon version 0.13.0 in mapping-based mode with mapping validation^47^. Read counts were used as the input for differential expression analysis using the Bioconductor package edgeR version 3.24.3^48^. For the time-course data analysis, one-way ANOVA-like testing was performed using the glmQLFTest function in edgeR with an FDR cutoff of 0.05. For the comparative data analysis, the quantile-adjusted conditional maximum-likelihood method was performed using the exactTest function in edgeR with an FDR cutoff of 0.05. GO and KEGG enrichment analyses were performed using the Bioconductor package clusterProfiler version 3.10.14^49^. Heatmaps and Venn diagrams were drawn by using the R package pheatmap version 1.0.12 and Venn version 1.7, respectively. Coexpression networks were constructed using the R package WGCNA version 1.68^50^ and visualized using Cytoscape version 1.7.2^51^. The phylogenetic tree of the *LCY* gene family was constructed with *CCS* genes as the outgroup among four eudicots (*Arabidopsis thaliana*, *Brassica napus*, *Populus trichocarpa*, and *Vitis vinifera*), four monocots (*Oryza sativa*, *Sorghum bicolor*, *Zea mays*, and *Brachypodium distachyon*), three magnoliids (*L. chinense*, *L. tulipifera*, and *Cinnamomum kanehirae*), one basal angiosperm (*Amborella trichopoda*), and one lycophyte (*Selaginella moellendorffii*) using RAxML version 8.2.12^52^.

### Quantitative real-time PCR

The transcript abundance of 11 carotenoid biosynthesis genes in the tuliptree petal band during flower development was quantified using qRT-PCR and the 2^−ΔΔCT^ method. Data were collected from three biological repeats. The actin gene was used as the reference gene. The primer sequences are listed in Supplementary Table 15. The *ε-LCY* gene (*Lchi18362*) in the *L. chinense* genome was almost identical to *Cluster-30529.103712*. We also used the primer sequences of *Cluster-30529.103712* to quantify the relative expression of the *ε-LCY* gene in the *L. chinense* petal area corresponding to the petal band of *L. tulipifera* during flower development.

### Reporting summary

Further information on research design is available in the Nature Research Reporting Summary linked to this article.

## Supplementary information

Reporting Summary

Supplementary Figures 1-23

Supplementary Table 1

Supplementary Table 2

Supplementary Table 3

Supplementary Table 4

Supplementary Table 5

Supplementary Table 6

Supplementary Table 7

Supplementary Table 8

Supplementary Table 9

Supplementary Table 10

Supplementary Table 11

Supplementary Table 12

Supplementary Table 13

Supplementary Table 14

Supplementary Table 15

## Data Availability

Raw reads have been deposited as a BioProject under accession PRJNA524246. Coding sequences, protein sequences, and annotation files are available at 10.6084/m9.figshare.11674020.v1.
