# Supplementary material for: Transcriptome analysis and metabolic profiling reveal the key role of carotenoids in the petal coloration of Liriodendron tulipifera
Source: Hortic Res. 2020 May 1;7:70. doi: 10.1038/s41438-020-0287-3 (PMC7193617; doi:10.1038/s41438-020-0287-3)
Supplement: Supplementary file 2 — Supplementary Figures 1-23 [file 41438_2020_287_MOESM2_ESM.pdf]

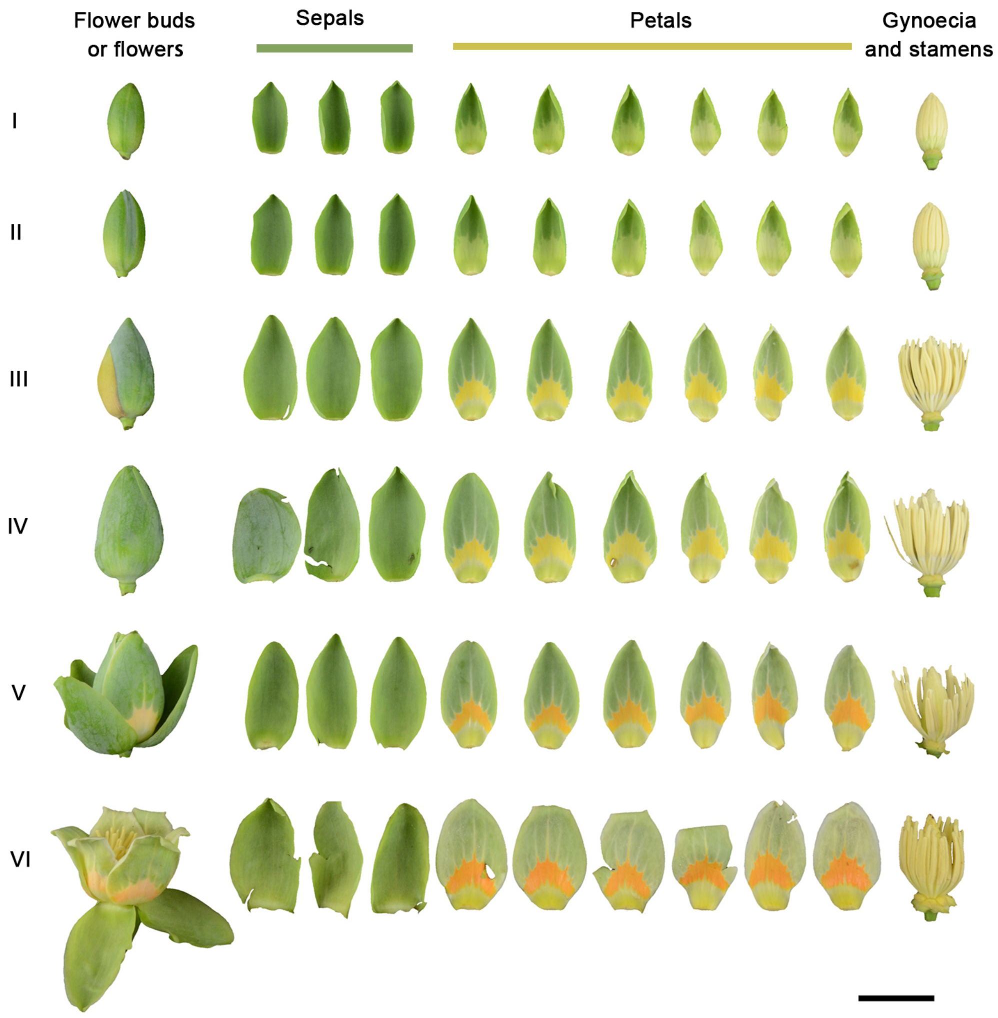

Supplementary Figure 1. Sample and developmental stage selection. We first observed and selected six different developmental stages based on the external characteristics of flower buds or flowers (detailed descriptions are included in Supplementary Table 1). There is no obvious color change between stage I and II, stage III and IV; therefore, we selected petal samples at stage II, III, V, and VI (renumberd as S1P, S2P, S3P, and S4P) for both metabolome and transcriptome time-series analyses. In addition, we also conducted two comparative analyses; one is between petals (S3P) and sepals (S3S) at stage V and the other is between the lower side (S3PL) and upper side (S3PU) of petals at stage V.

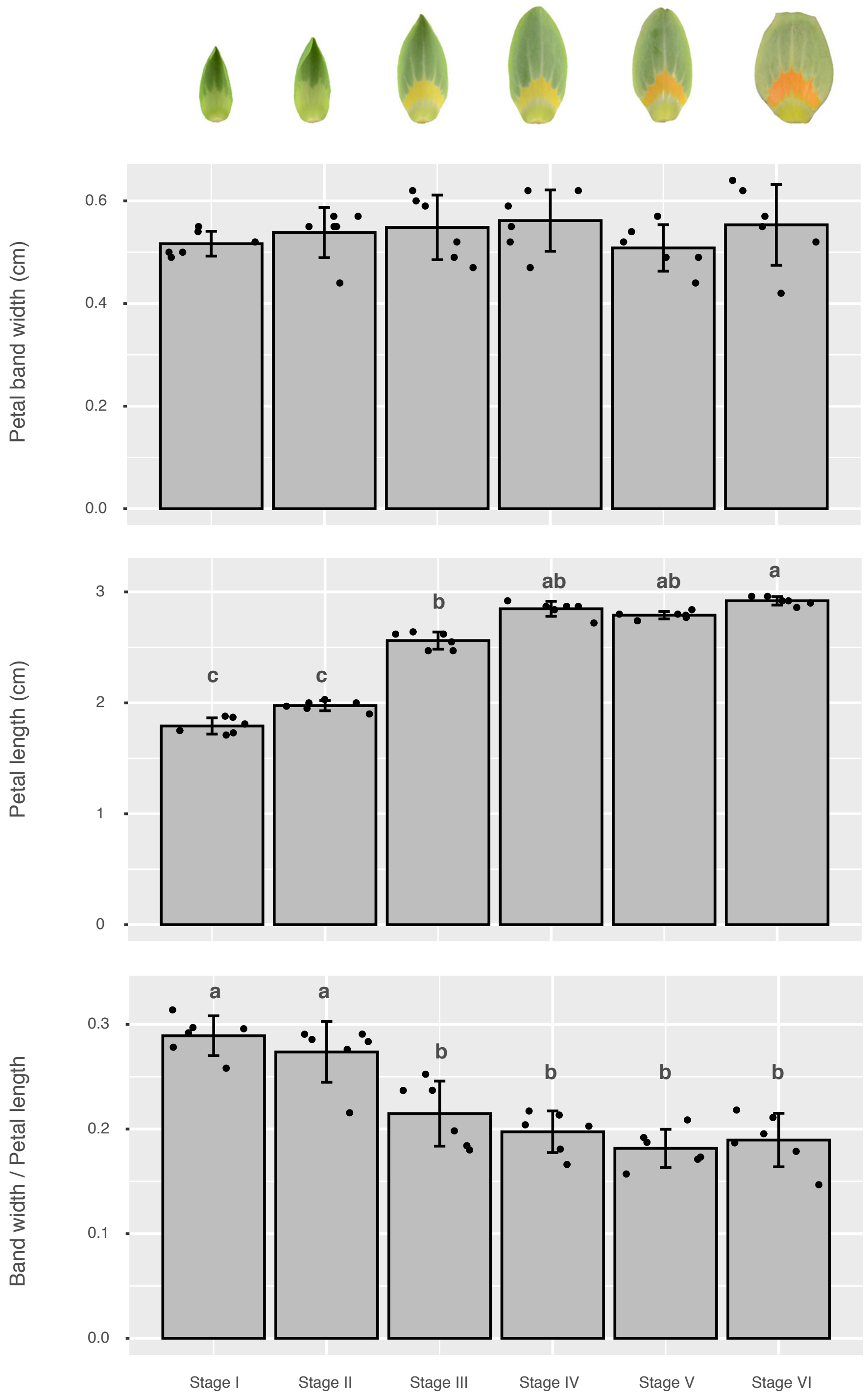

Supplementary Figure 2. Petal band and length dynamics during the petal development in *L. tulipifera*. The width of petal band and petal length were both measured along the middle vein. Multiple comparisons were performed using the function LSD.test which is implemented in the R package *agricolae*.

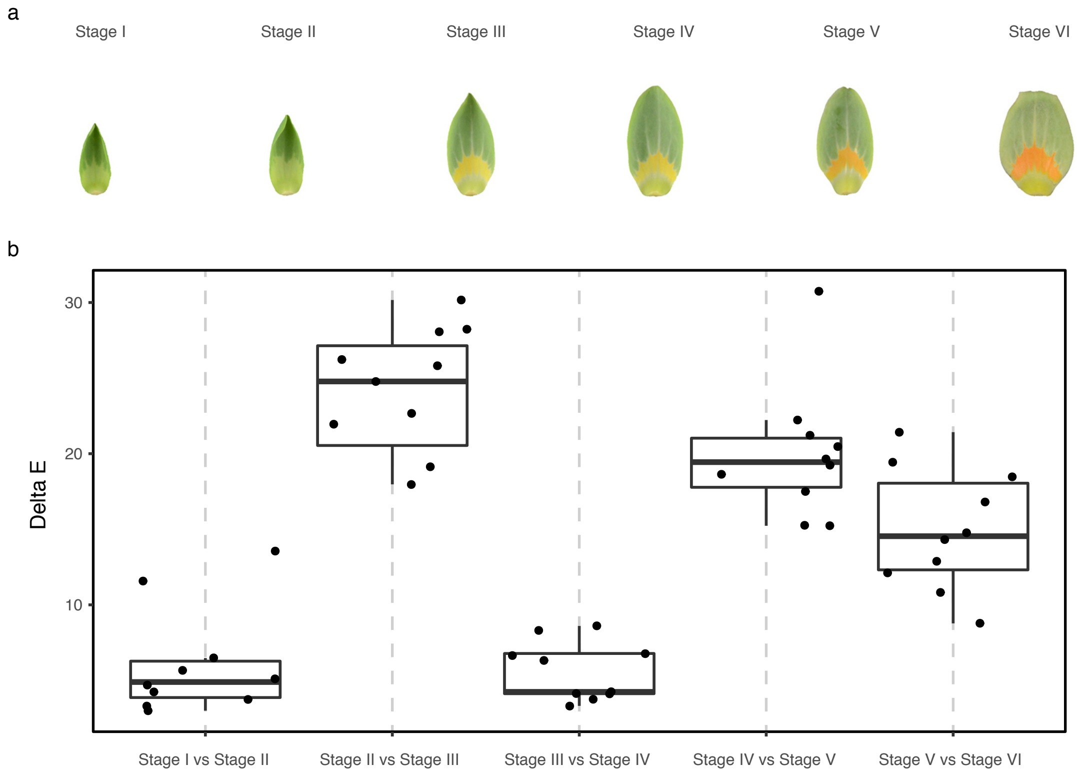

Supplementary Figure 3. Color changes during the petal development. (a) Representative petals from different developmental stages. (b) Quantification of color change on the band of *L. tulipifera* petals using the Delta E algorithm.

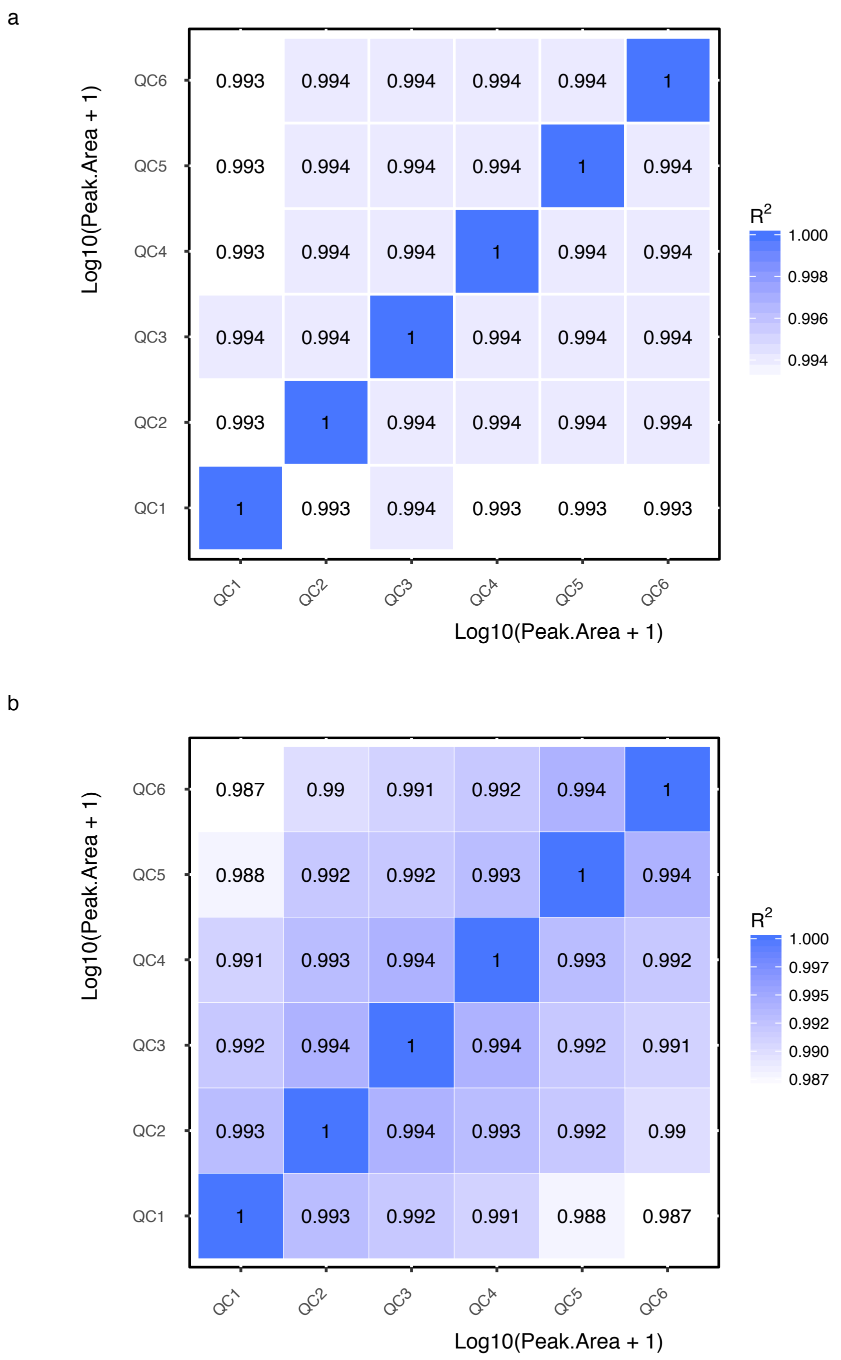

Supplementary Figure 4. Pearson correlation between QC samples. (a) Pearson correlation between QC samples in the positive ionization mode. (b) Pearson correlation between QC samples in the negative ionization mode.

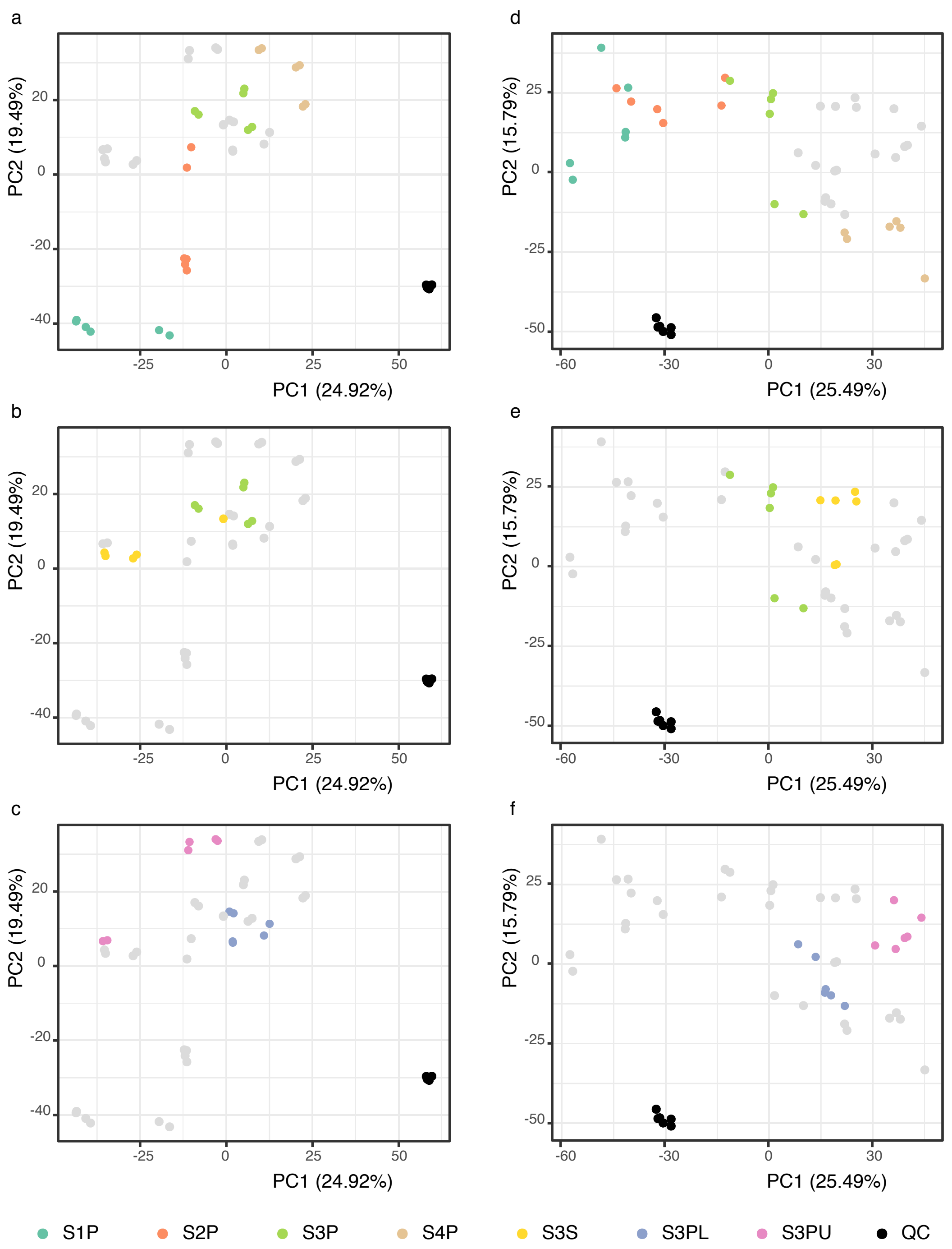

Supplementary Figure 5. PCA plots for all samples. PC1 and PC2 explain 24.92% and 19.49%, 25.49% and 15.79% in the positive (a-c) and negative (d-f) ionization mode, respectively. Each stage or tissue has six replicates and is represented by dots in different colors. In both positive and negative ionization mode, QC samples are all clustered together very tightly and separated from all the other samples. In addition, a clear distinction between groups can be seen in the time-course experiment (a and d) and both comparative analyses, i.e., petals and sepals (b and e) and the lower side and upper side of petals (c and f) in the stage III.

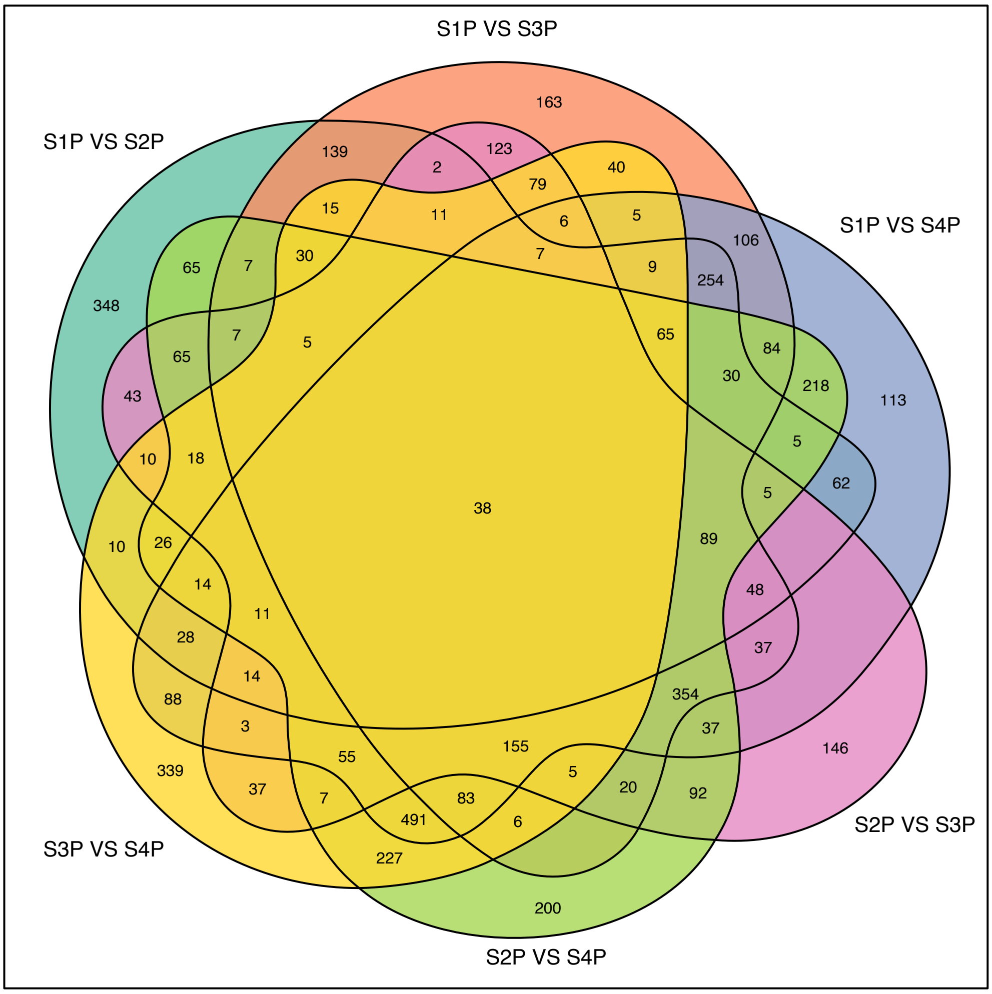

Supplementary Figure 6. Venn diagram showing the metabolites that are significantly different between different petal developmental stages. Six pairwise comparisons were performed to identified metabolites that are significant differential between at least two *Liriodendron* petal developmental stages.

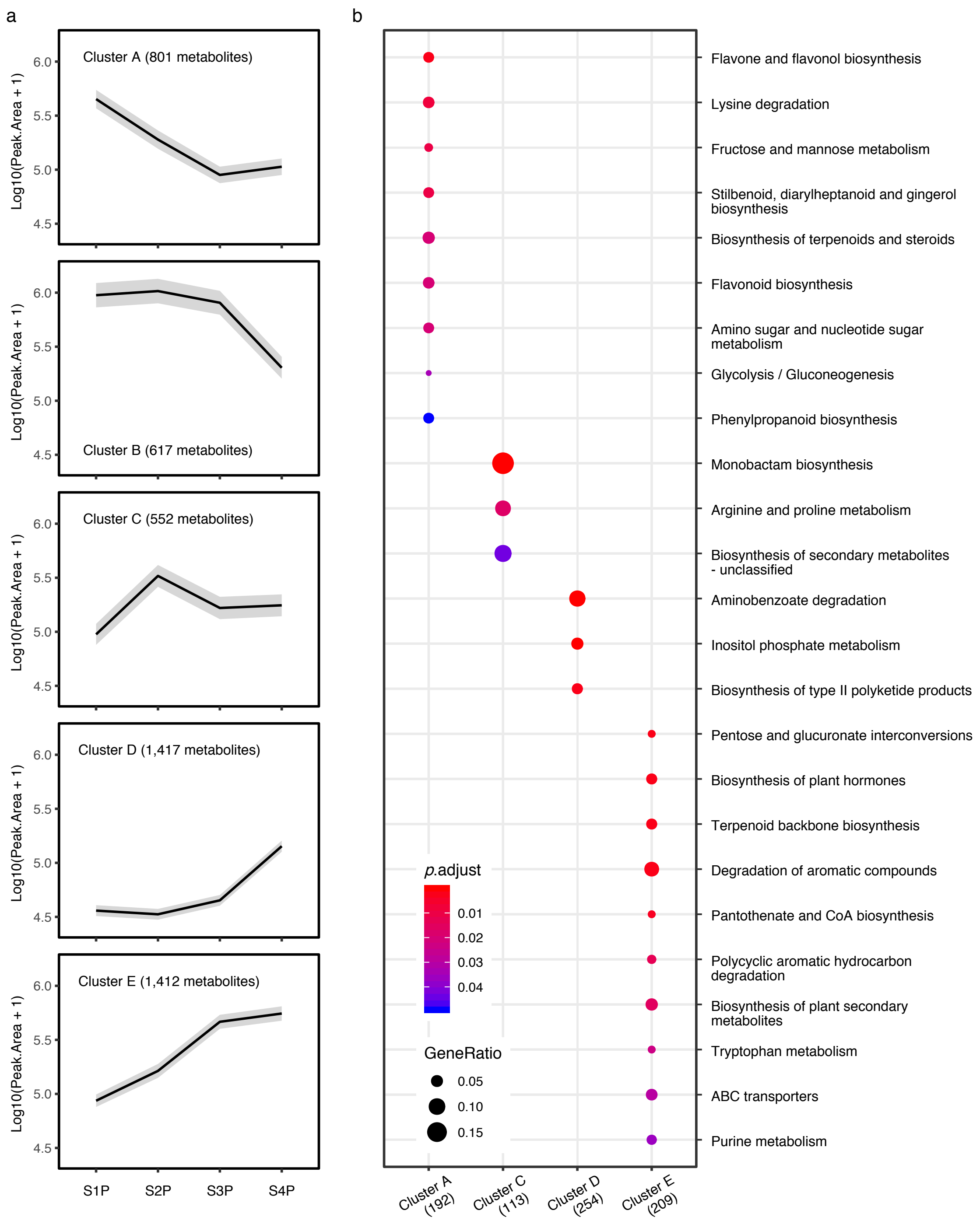

Supplementary Figure 7. K-mean clustering of significantly changed metabolites during different *Liriodendron* petal developmental stages and KEGG enrichment analysis. (a) Means of metabolite cluster normalized content profiles in the time-course dataset. The grey shade area represents the 95% confidence interval. (b) Comparative KEGG pathway enrichment analysis among five clusters.

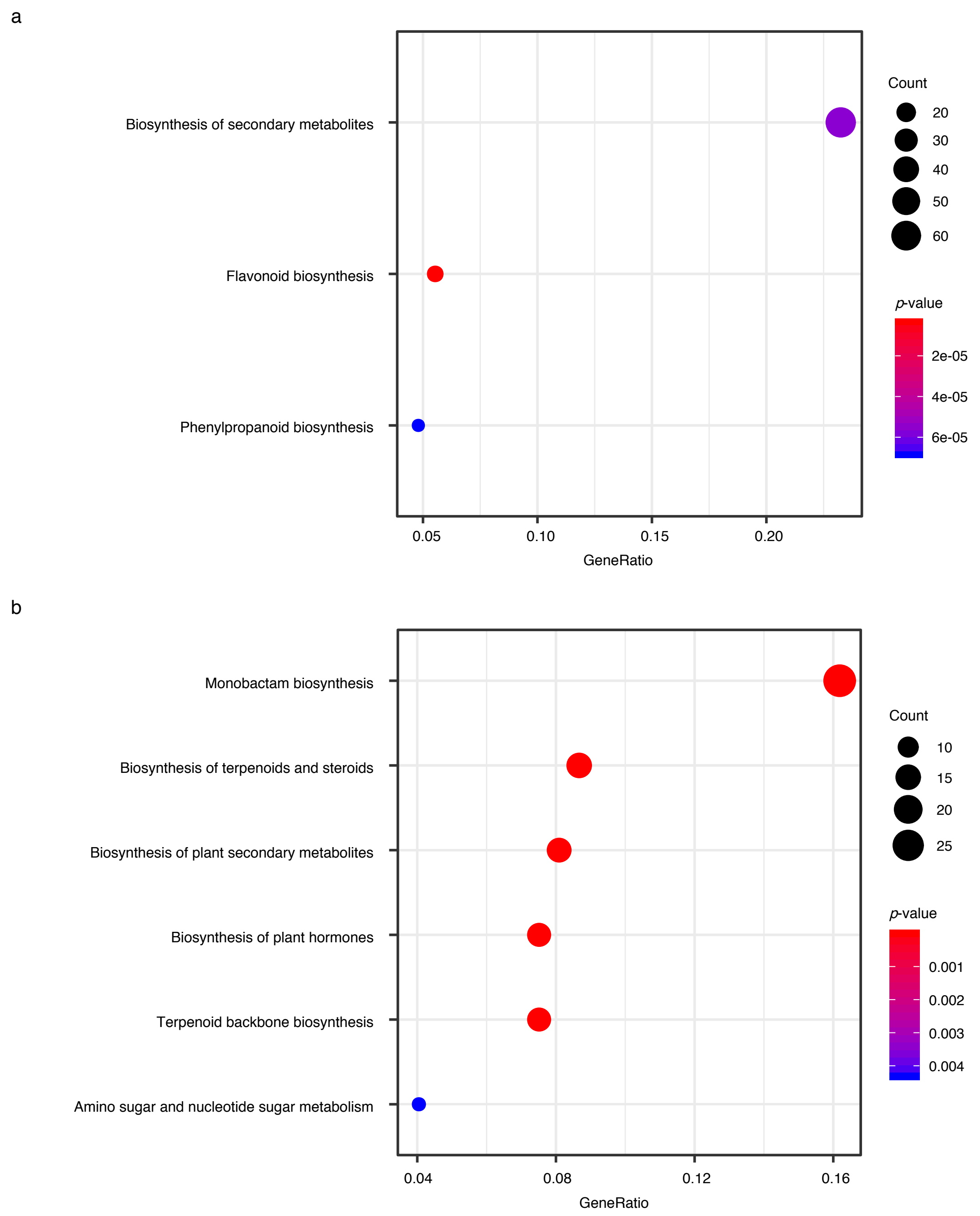

Supplementary Figure 8. KEGG enrichment analysis for two comparative datasets. (a) KEGG enrichment analysis for the metabolites that are significantly different between petals and sepals. (b) KEGG enrichment analysis for the metabolites that are significantly different between the lower and upper sides of petals.

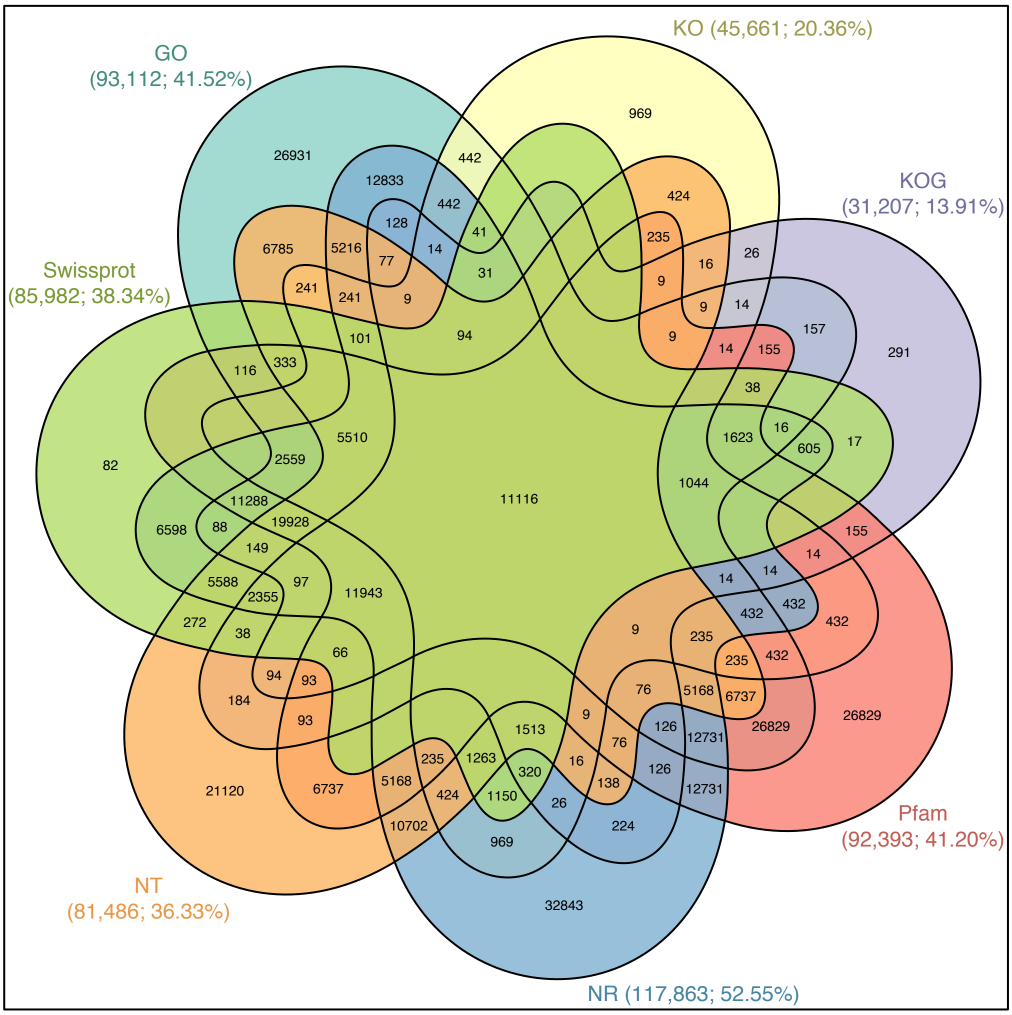

Supplementary Figure 9. Transcriptome functional annotation. All transcripts were annotated through homology based sequence comparison across seven databases, i.e., NCBI non-redundant protein sequences (NR), NCBI nucleotide sequences (NT), Pfam, eukaryotic orthologous groups (KOG), Swiss-prot, Kyoto Encyclopedia of Genes and Genomes (KEGG) and Gene Ontology (GO). The numbers and percentages in brackets are annotated transcripts in one database and proportions they accounted for of the total transcripts.

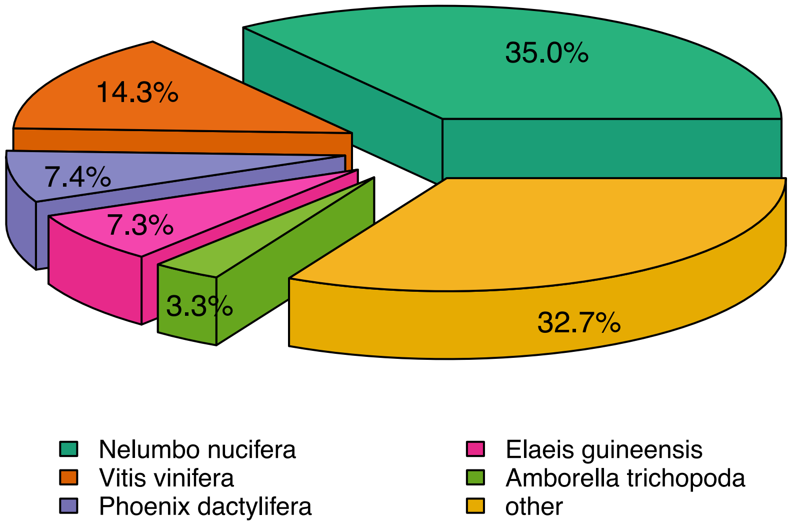

Supplementary Figure 10. The BLASTX hit species distribution in the NR database. This chart showed that the different species to which most transcripts were aligned during the BLASTX step.

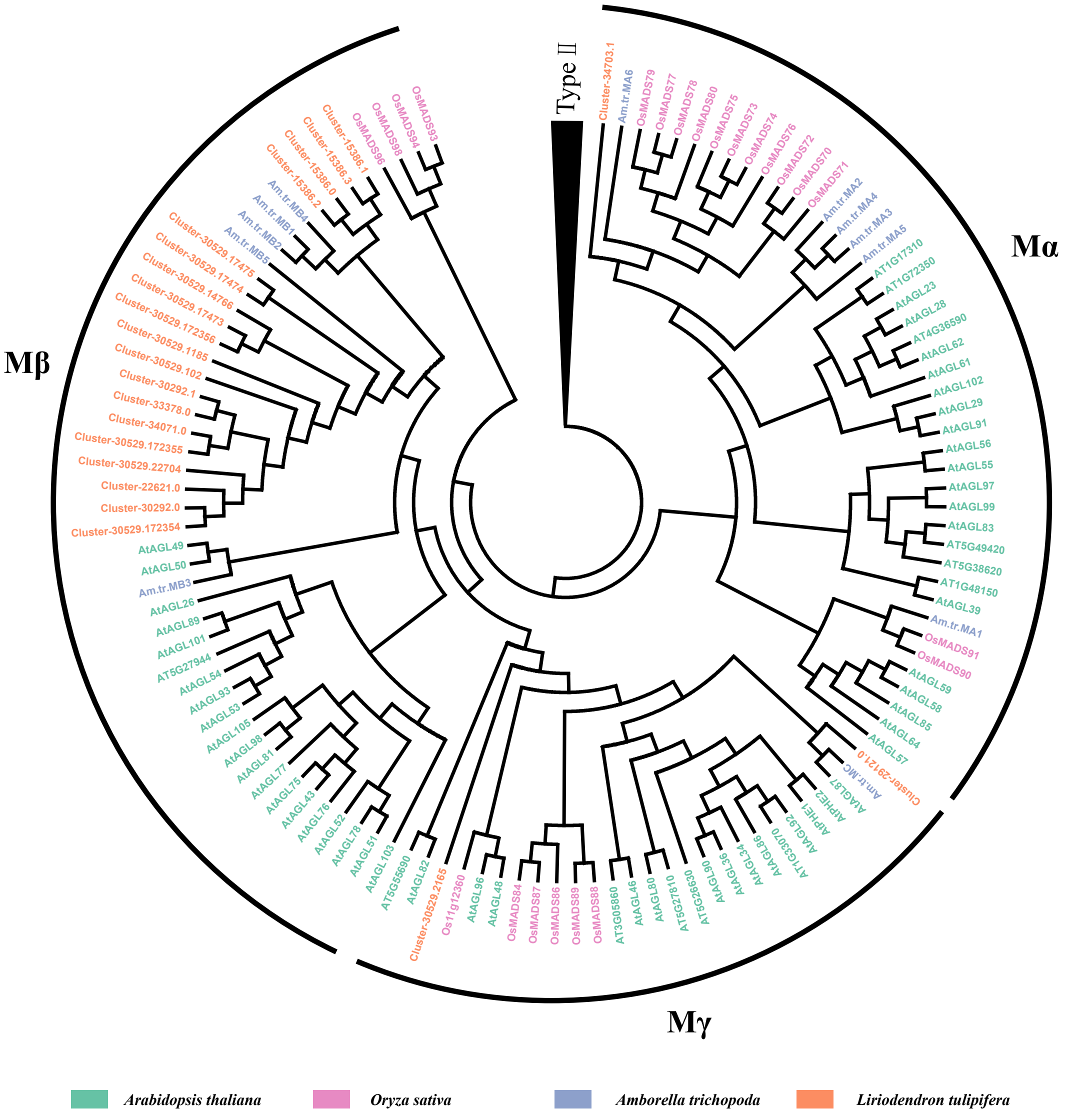

Supplementary Figure 11. Phylogenetic tree of type I MADS genes. The tree was constructed using the ML method based on the complete amino acid sequences and rooted by type II MADS genes.

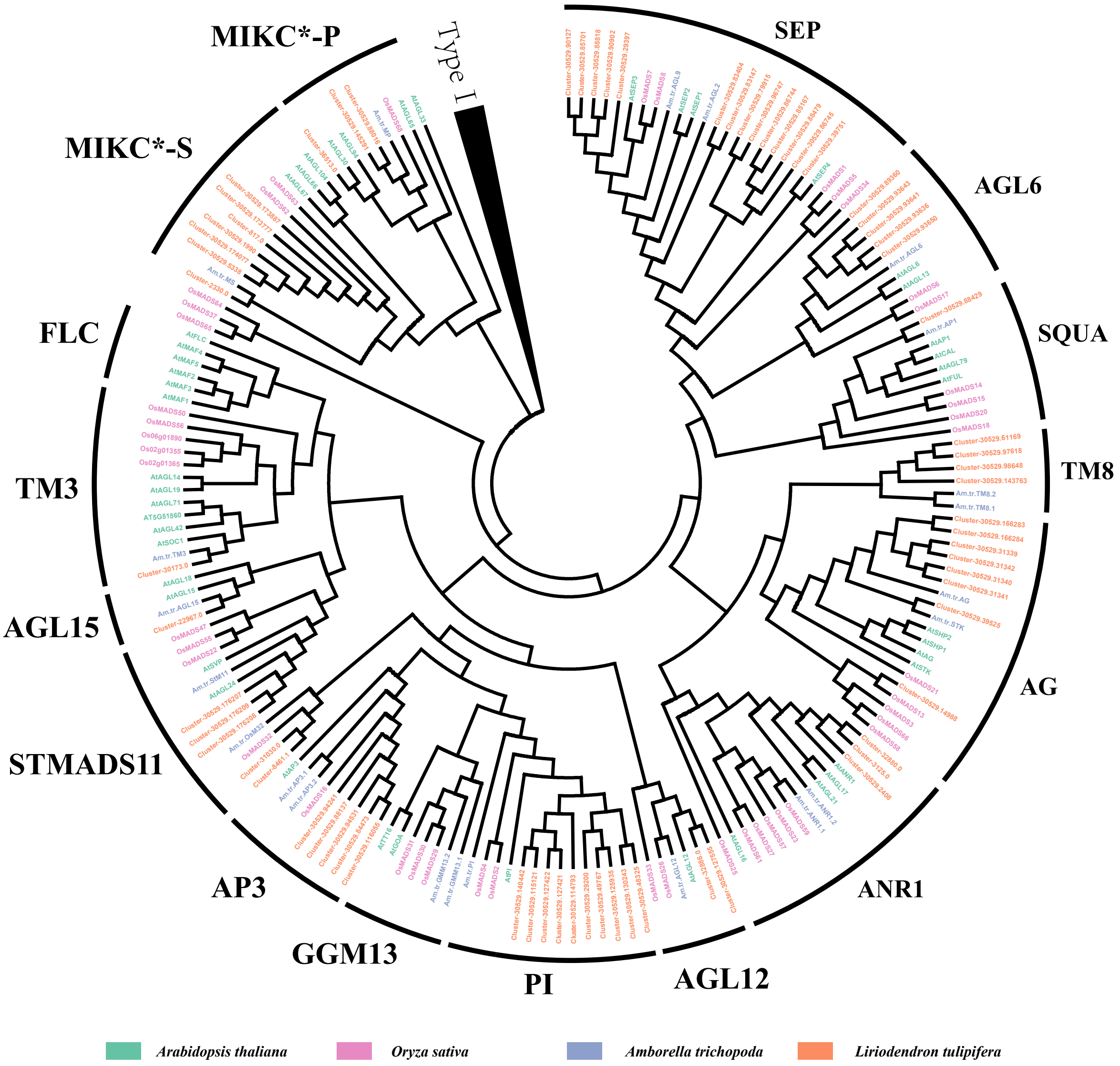

Supplementary Figure 12. Phylogenetic tree of type II MADS genes. The tree was constructed using the ML method based on the complete amino acid sequences and rooted by type I MADS genes.

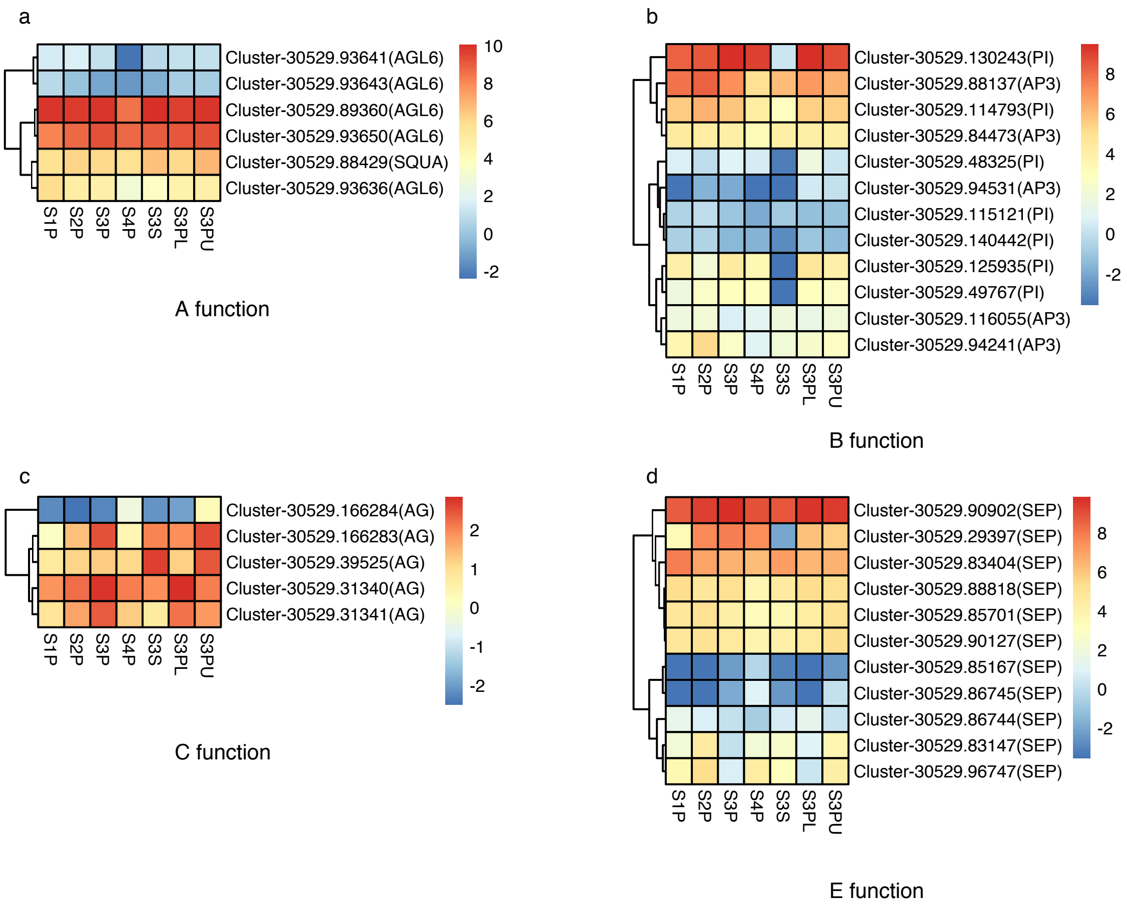

Supplementary Figure 13. The expression of ABCE model genes across the tuliptree flower development.

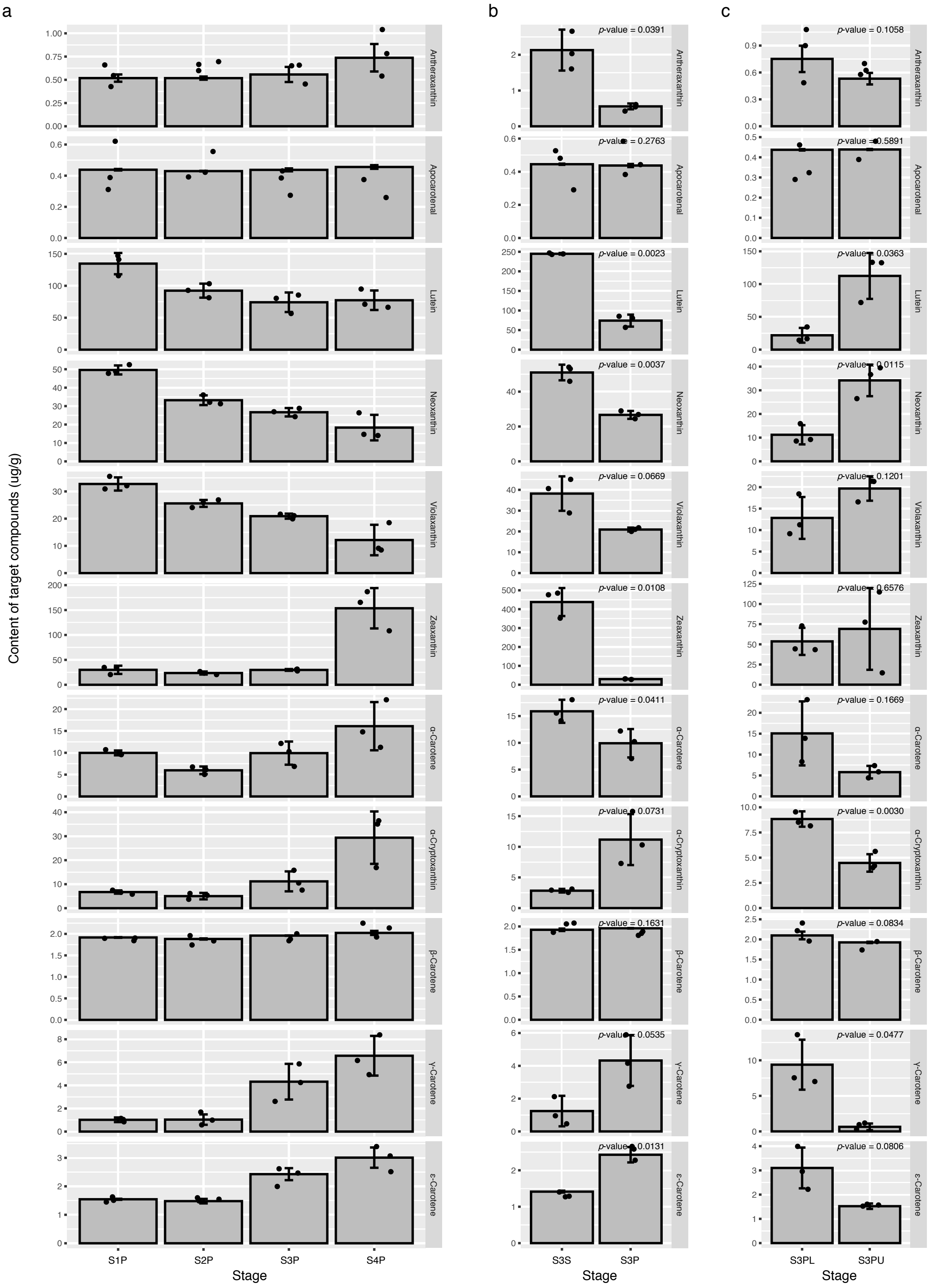

Supplementary Figure 14. The absolute quantification of 11 compounds from the carotenoid biosynthesis pathway. (a) The content dynamics of these compounds during the petal development. (b) Comparison of compound content between petals and sepals. (c) Comparison of compound content between the lower and upper sides of petals.

a

b

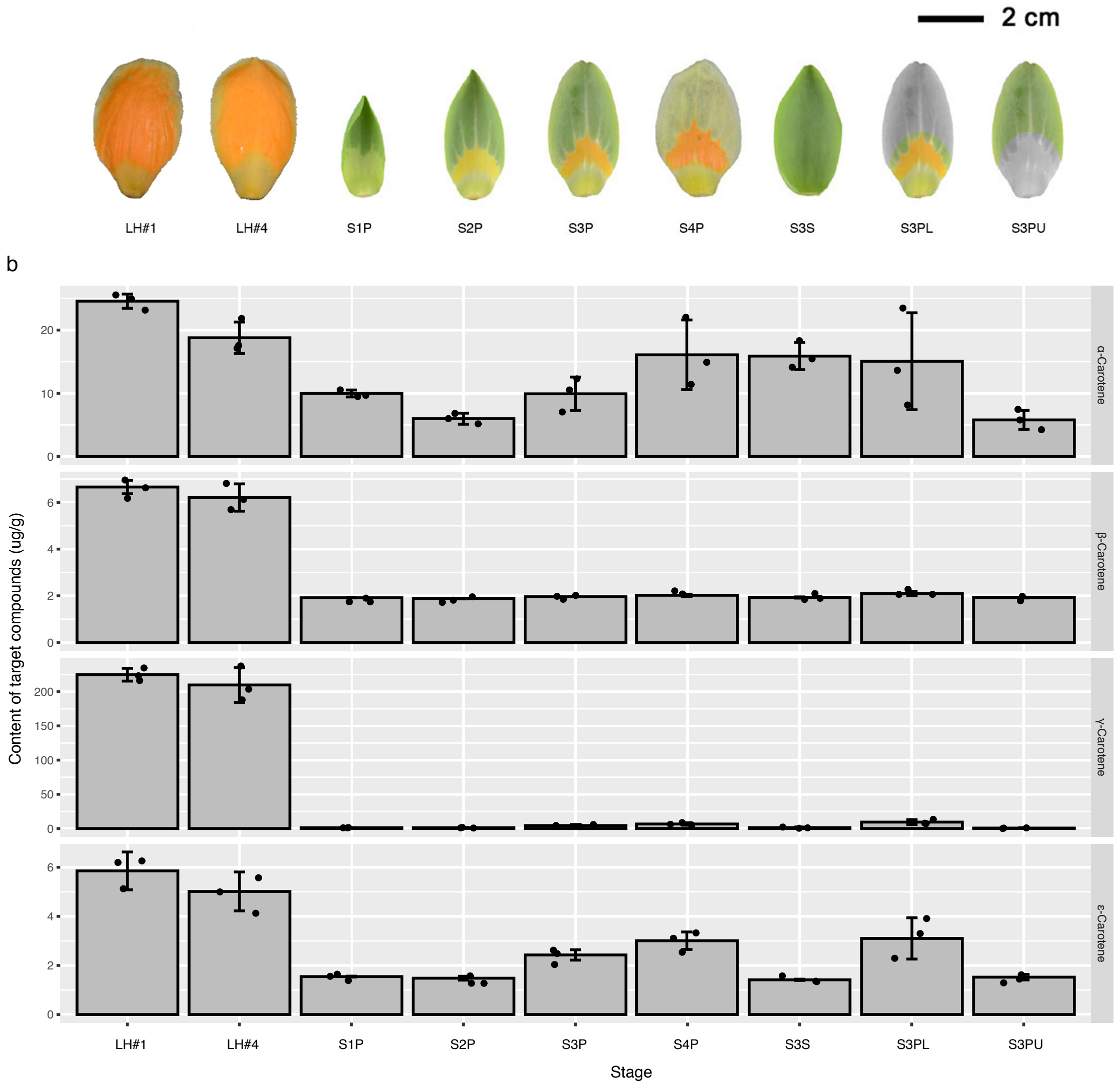

Supplementary Figure 15. The content of four carotenes in petals of *Liriodendron* hybrids. (a) Representative petals or sepals from different developmental stages and individuals. (b) The absolute content of four carotenes across different developmental stages, tissues and individuals.

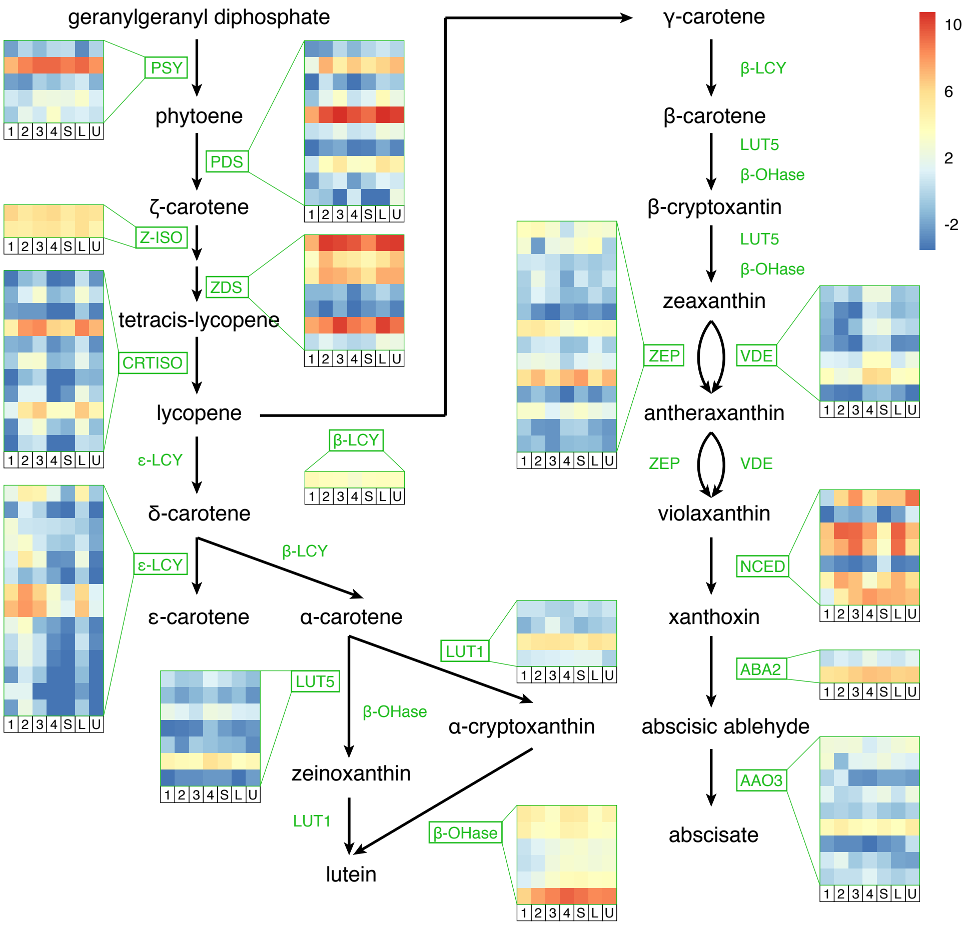

Supplementary Figure 16. Expression profiles of carotenoid biosynthesis genes during the *L. tulipifera* flower development. Absolute expression profiles (blue-yellow-red scale) of genes implicated in petal coloration. 1, S1P; 2, S2P; 3, S3P; 4, S4P; P, S3P; S, S3S; L, S3PL; U, S3PU; PSY, phytoene synthase; PDS, phytoene desaturase; Z-ISO, ζ-carotene isomerase; ZDS, ζ-carotene desaturase; CRTISO, carotenoid isomerase; ε-LCY, lycopene ε-cyclase; β-LCY, lycopene β-cyclase; LUT5, β-ring hydroxylase; β-OHase, β-carotene hydroxylase; LUT1, carotene ε-monooxygenase; ZEP, zeaxanthin epoxidase; VDE, violaxanthin de-epoxidase; NCED, 9-cis-epoxycarotenoid dioxygenase; ABA2, xanthoxin dehydrogenase; AAO3, abscisic-aldehyde oxidase.

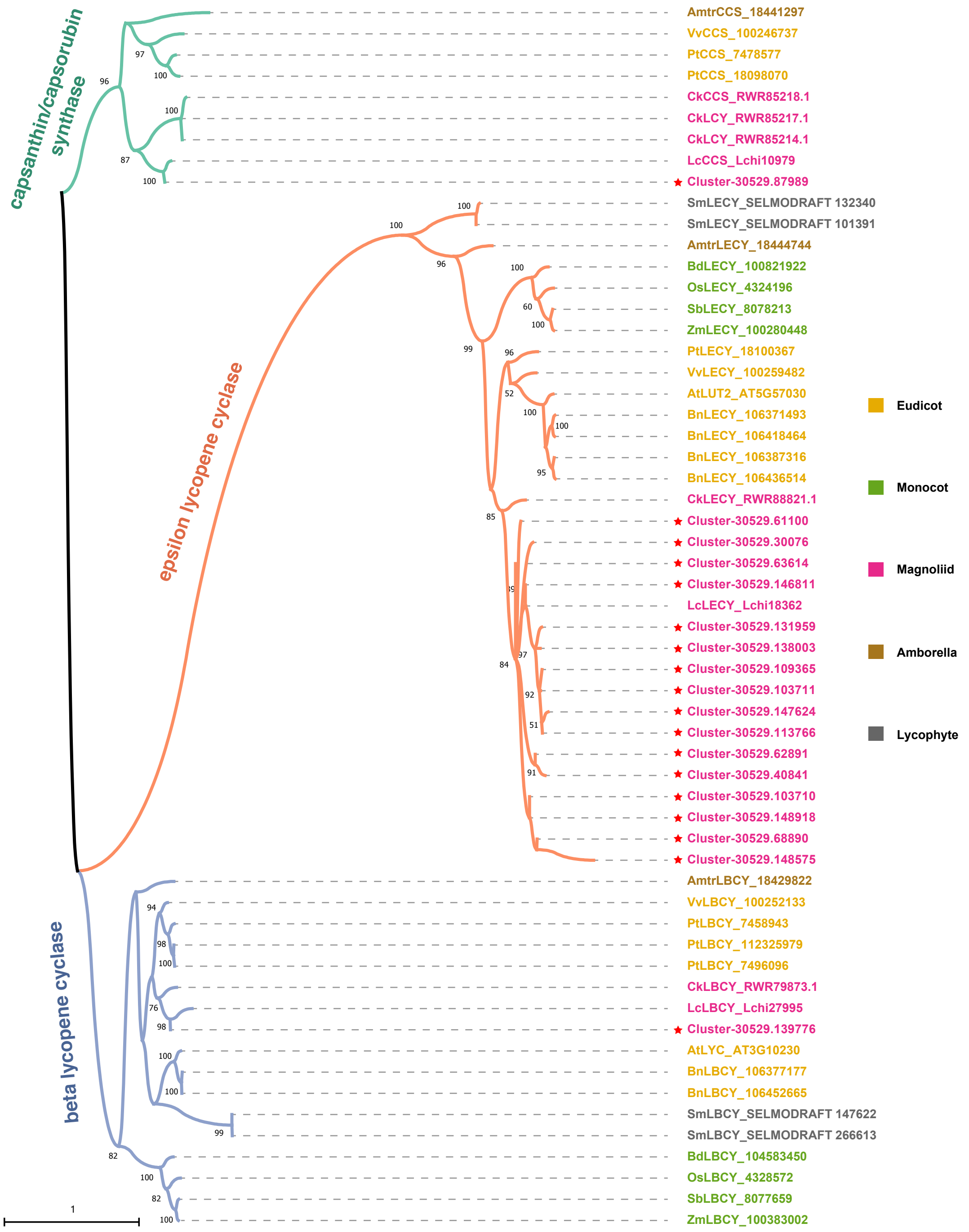

Supplementary Figure 17. Phylogenetic tree of *LCY* genes constructed based on 13 plant species. All genes were obtained from the KEGG database with the entry ID next to the gene name, except for those of *L. chinense* and C. *kanehirae* which were extracted from their genome annotations. The phylogenetic tree was constructed using ML method with *CCS* genes being the outgroup. At, *Arabidopsis thaliana*; Bn, *Brassica napus*; Pt, *Populus trichocarpa*; Vv, *Vitis vinifera*; Os, *Oryza sativa*; Sb, *Sorghum bicolor*; Zm, *Zea mays*; Bd, *Brachypodium distachyon*; Lc, *Liriodendron chinense*; Ck, *Cinnamomum kanehirae*; Amtr, *Amborella trichopoda*; Sm, *Selaginella moellendorffii*.

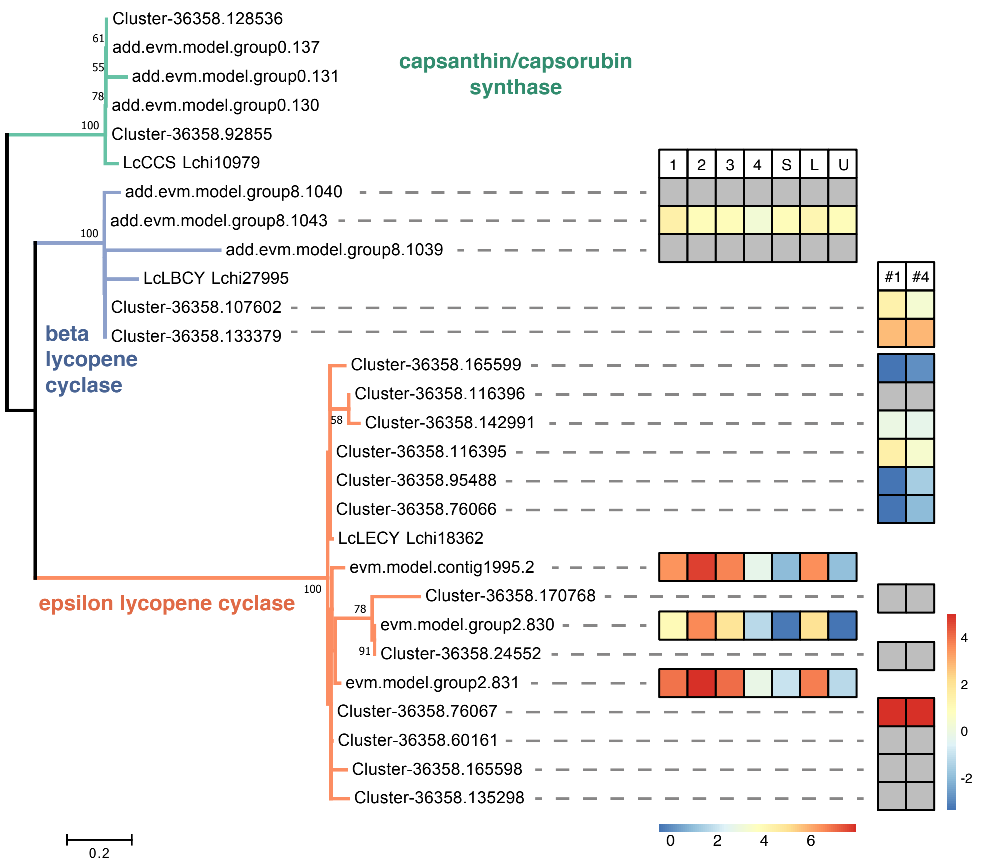

Supplementary Figure 18. Phylogenetic tree of *LCY* genes constructed based on three *Liriodendron* species. All gene sequences being used in the phylogenetic tree construction were included in Supplementary Table 11. All amino acid sequences were aligned using Clustal Omega and trimmed using TrimAl. Then, the *LCY* gene tree was constructed using RAxML with *CCS* genes being the outgroup. The heat maps came from two datasets, one is petal and sepal transcriptomes from *L. tulipifera* as presented in this study and the other is petal transcriptomes from *Liriodendron* hybrid (unpublished data).

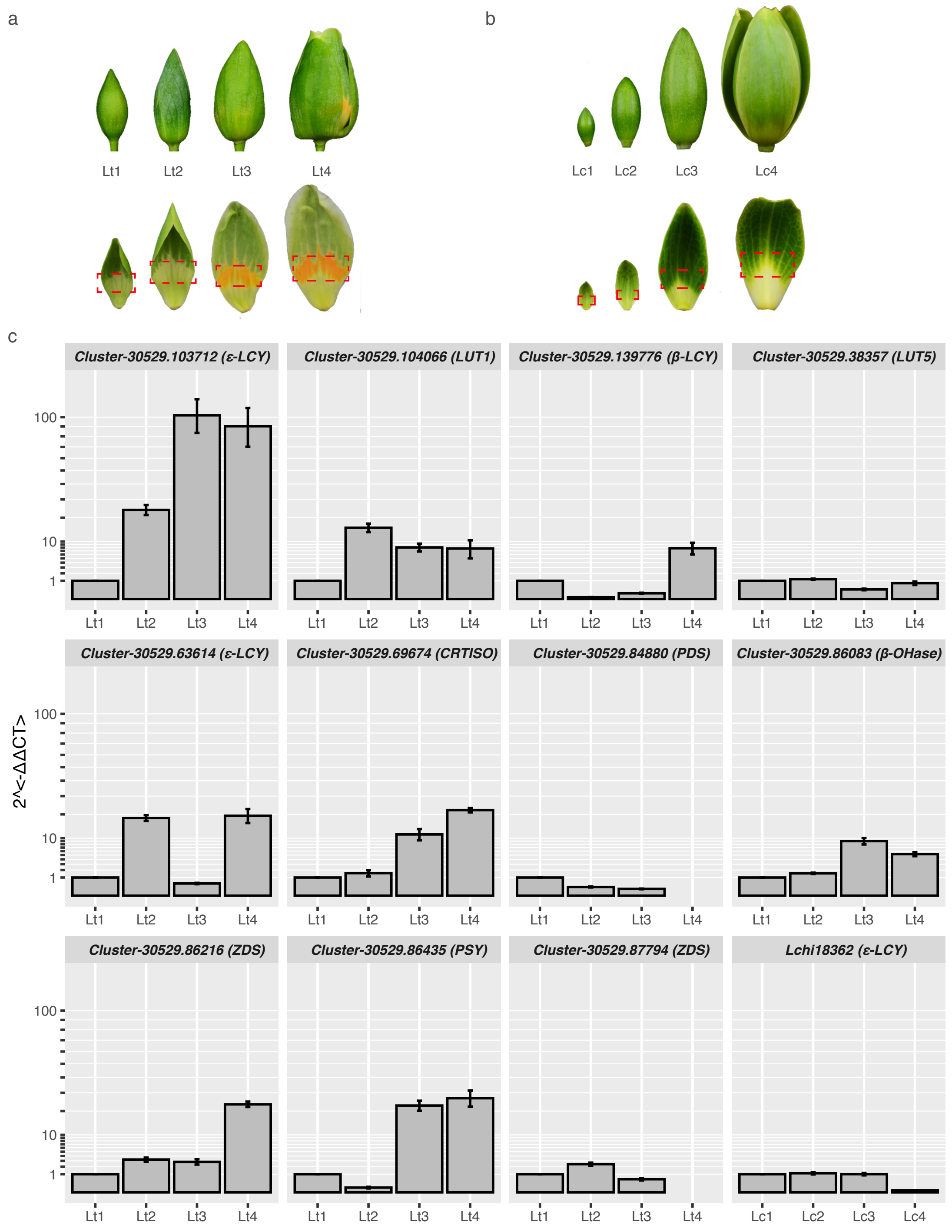

Supplementary Figure 19. Transcript abundance measured by qRT-PCR analysis. The flower buds and petals of *L. tulipifera* (a) and *L. chinense* (b) were shown in four different petal developmental stages. The red-dashed boxes indicated the petal area being used for qRT-PCR experiments. (c) The relative gene expression of 11 carotenoid biosynthesis genes in *L. tulipifera* and the only one *ε-LCY* gene in *L. chinense*. For each gene, the relative expression was compared to the first stages. The relative expression of *PDS* and *ZDS* at the last stage were not plotted due to the missing values.

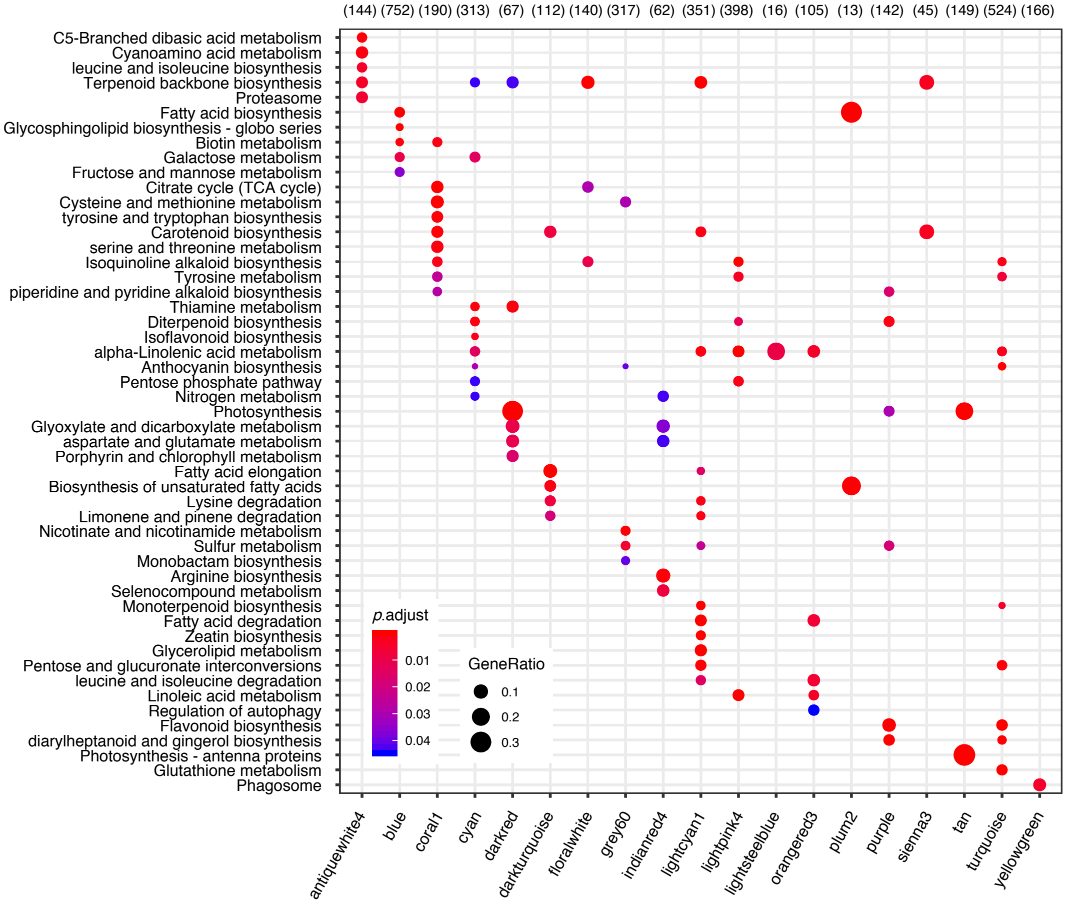

Supplementary Figure 20. KEGG pathway enrichment analysis of 26 modules constructed from WGCNA.

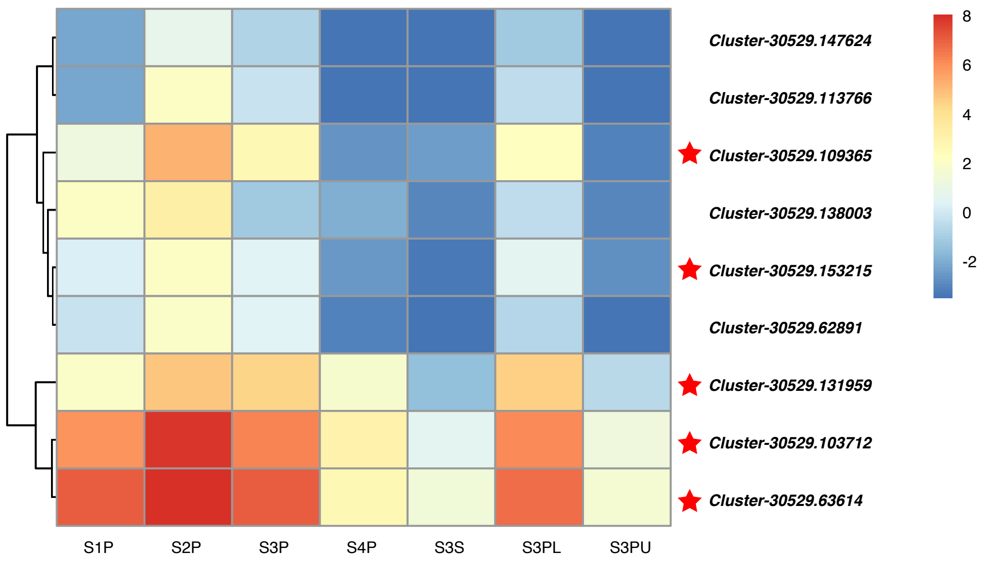

Supplementary Figure 21. An expression heatmap of nine *ε-LCY* unigenes contained in the coral1 module. Genes that were significantly up-regulated in petals and the lower side of petals compared to petals and the upper side of petals, respectively, were indicated with red stars.

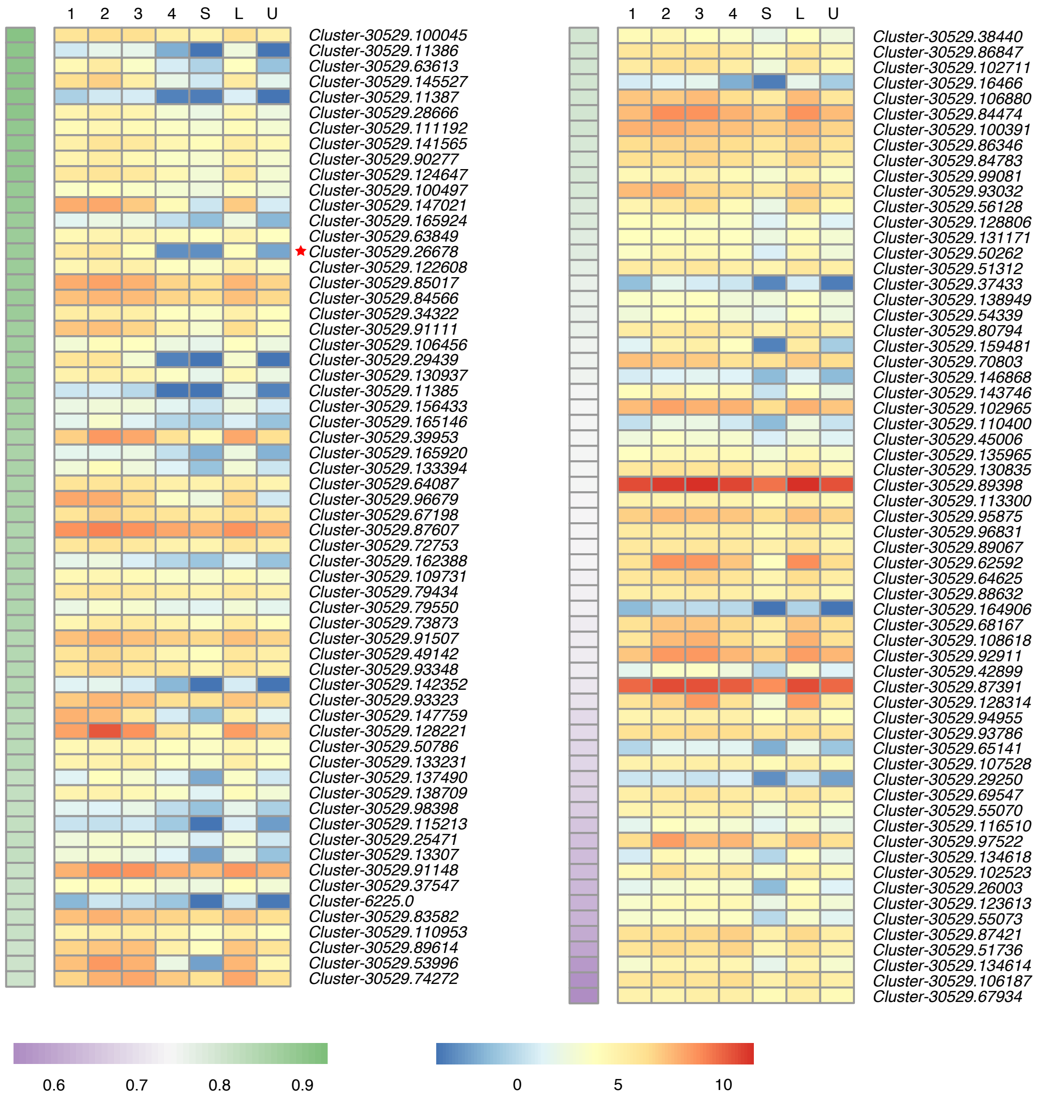

Supplementary Figure 22. The gene expression relationship between five *ε-LCY* unigenes and 125 co-expressed unigenes. Average expression correlation (purple-white-green scale) between five *ε-LCY* unigenes and 125 co-expressed unigenes and absolute expression profiles (blue-yellow-red scale) of these 125 co-expressed unigenes were plotted in a heatmap. The unigene which was annotated as a member of bHLH transcription factor family was indicated with a red star. 1, S1P; 2, S2P; 3, S3P; 4, S4P; P, S3P; S, S3S; L, S3PL; U, S3PU.

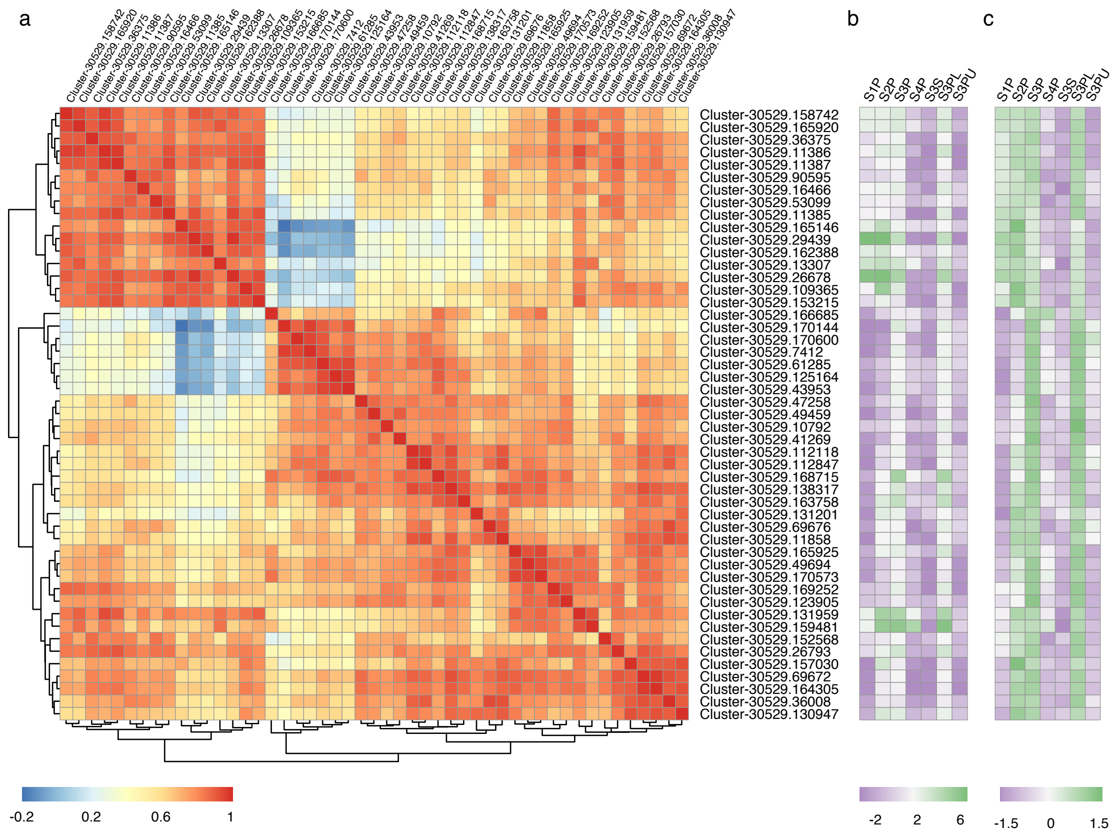

Supplementary Figure 23. 49 key unigenes during the petal band coloration in *L. tulipifera*. (a) The Pearson correlation of gene expression among these 49 unigenes. (b) Absolute gene expression profiles. (c) Relative gene expression profiles.
